# Genomic insights from the first chromosome-scale assemblies of oat (*Avena* spp.) diploid species

**DOI:** 10.1186/s12915-019-0712-y

**Published:** 2019-11-22

**Authors:** Peter J. Maughan, Rebekah Lee, Rachel Walstead, Robert J. Vickerstaff, Melissa C. Fogarty, Cory R. Brouwer, Robert R. Reid, Jeremy J. Jay, Wubishet A. Bekele, Eric W. Jackson, Nicholas A. Tinker, Tim Langdon, Jessica A. Schlueter, Eric N. Jellen

**Affiliations:** 1 Department of Plant & Wildlife Sciences, Brigham Young University, 4105 LSB, Provo, UT 84602 USA; 2 University of North Carolina at Charlotte, Charlotte, NC 28223 USA; 3 IBERS, Aberystwyth University, Aberystwyth, Wales UK; 4 Agriculture and Agri-Food Canada, Ottawa, ON K1A 0C6 Canada; 5 25:2 Solutions, Rockford, MN USA

**Keywords:** Aveninae, *Avena*, Oat, Hi-C, Flowering time, Crown rust resistance, Polyploidy

## Abstract

**Background:**

Cultivated hexaploid oat (Common oat; *Avena sativa*) has held a significant place within the global crop community for centuries; although its cultivation has decreased over the past century, its nutritional benefits have garnered increased interest for human consumption. We report the development of fully annotated, chromosome-scale assemblies for the extant progenitor species of the A_s_- and C_p_-subgenomes, *Avena atlantica* and *Avena eriantha* respectively. The diploid *Avena* species serve as important genetic resources for improving common oat’s adaptive and food quality characteristics.

**Results:**

The *A. atlantica* and *A. eriantha* genome assemblies span 3.69 and 3.78 Gb with an N50 of 513 and 535 Mb, respectively. Annotation of the genomes, using sequenced transcriptomes, identified ~ 50,000 gene models in each species—including 2965 resistance gene analogs across both species. Analysis of these assemblies classified much of each genome as repetitive sequence (~ 83%), including species-specific, centromeric-specific, and telomeric-specific repeats. LTR retrotransposons make up most of the classified elements. Genome-wide syntenic comparisons with other members of the Pooideae revealed orthologous relationships, while comparisons with genetic maps from common oat clarified subgenome origins for each of the 21 hexaploid linkage groups. The utility of the diploid genomes was demonstrated by identifying putative candidate genes for flowering time (HD3A) and crown rust resistance (*Pc*91). We also investigate the phylogenetic relationships among other A- and C-genome *Avena* species.

**Conclusions:**

The genomes we report here are the first chromosome-scale assemblies for the tribe Poeae, subtribe Aveninae. Our analyses provide important insight into the evolution and complexity of common hexaploid oat, including subgenome origin, homoeologous relationships, and major intra- and intergenomic rearrangements. They also provide the annotation framework needed to accelerate gene discovery and plant breeding.

## Background

Oat (*Avena sativa* L.) is a nutritionally important crop throughout the world. It is ranked 6th in world cereal production [1], and while its primary use continues to be as a livestock feed, its uses as a human food and for cosmetics continue to increase [2]. Among the many nutritional benefits of oat are its high levels of calcium, β-glucan soluble fiber [3–6], and high-quality oil and protein [7, 8]. Oat seed contains no gluten and only low levels of gluten-related prolamins and therefore is a healthy diet alternative for many people who cannot tolerate dietary gluten. Oat is high in polyphenolic avenanthramides having antioxidant, anti-inflammatory, and antiatherogenic properties [9]. Oat also contains two classes of saponins: avenacosides (sugars bound to steroids) and avenacins (sugars bound to triterpenoid), both of which have been shown to lower cholesterol, stimulate the immune system, and have anti-carcinogenic properties [7]. Oat also has many topical uses, as it has a soothing effect on skin and has been used to treat dry, itchy skin [10]; oat has also been shown to have sun-blocking properties [11], and it is often found in products to treat eczema, psoriasis, and other skin conditions [12, 13].

Common oat (*A. sativa*) and red oat (*A. byzantina* C. Koch) are allohexaploids (2*n* = 6*x* = 42, AACCDD subgenomes) belonging to the Poeae Tribe of the Poaceae [14] and are thought to have been domesticated from wild-weedy *A. sterilis* L. [15], a species that arose from hybridization between a CCDD allotetraploid closely related to modern *A. insularis* Ladiz. and an A_s_A_s_ diploid [16]. Several variants of the A-subgenome diploids exist (A_c_, A_d_, A_l_, A_p_, and A_s_ [17];) and are known to harbor several genetic features of significance, including major crown rust resistance genes that have been transferred into hexaploid oat cultivars [18, 19]. The A-genome diploids have also been identified as potential gene sources for improving soluble fiber and protein [20]. The A-subgenome is also part of a major intergenomic translocation (7C-17A) in *A. sativa-A. sterilis* that has been associated with adaptation to winter hardiness—key elements in oat production that likely contributed to the plant’s ability to shift from Mediterranean winter ecology to Eurasian spring-summer cultivation [21].

The C-subgenome chromosomes have a high amount of diffuse heterochromatin along their entirety [22]; this is a genetic feature not seen in the A and D chromosomes, where heterochromatin is localized and seemingly concentrated around the centromeres, at the telomeres, and in flanking secondary constrictions where rRNA genes are located. Among the important genetic features in the C-subgenome is a terminal translocation segment on the long arm of 21D which carries a putative *CSlF6c* locus that likely has a negative effect on seed soluble fiber content [23, 24]. Linkage has also been demonstrated between the chromosome 5C telomeric knob in allotetraploid *A. magna* Murphy et Terrell (CCDD subgenomes) and co-segregating genes controlling awn production and basal abscission layer formation which have been implicated in the domestication of common oat [25].

Despite the historical importance of oat and the renewed interest in its nutritional value, a complete genome sequence of oat has yet to be reported. Indeed, the *A. sativa* genome is large (> 12 Gb [26]), duplicated, complex, highly repetitive, and characterized by several major intra- and intergenomic rearrangements—making full genome assembly of the hexaploid difficult [27]. Here we report the development of fully annotated, chromosome-scale assemblies for the extant progenitor species of the A_s_- and C_p_-subgenomes, *A. atlantica* B.R.Baum & Fedak and *Avena eriantha* Durieu., respectively. Using these assemblies, we (i) identified and quantified repetitive element content in the genome, including centromeric and telomeric repeats, (ii) analyzed syntenic relationships with other cereal grains and homoeologous relationships within oat using consensus linkage maps [28], (iii) identified putative candidate genes for flowering time [29] and crown rust resistance [30] relative to recently published genome-wide association studies (GWAS), (iv) estimated the age of the evolutionary split between the A- and C-subgenomes using synonymous substitution rates (*K*_*s*_) analysis, and (v) examined the genetic diversity and phylogenetic relationship from a resequencing panel of 76 A- and C-genome *Avena* species.

## Results

### Whole-genome sequencing and assembly

We selected the *A. atlantica* accession Cc 7277 and the *A. eriantha* accession CN 19328 for whole-genome shotgun sequencing. Both accessions are highly inbred and phenotypically homogeneous and represent type accessions for their respective species. A total of 31,544,396 and 28,257,346 PacBio reads were generated across 122 (RSII and Sequel) and 54 (Sequel) SMRT cells generating a total of 325.9 (~84× coverage) and 276.6 (~71× coverage) Gb of sequence data for *A. atlantica* and *A. eriantha*, respectively. The longest reads for each species, 194,884 and 151,576 bp, came from the Sequel instrument. The N50 read length for *A. atlantica* and *A. eriantha* was 18,658 and 15,102 bp, respectively. In addition to PacBio sequencing, a total of 192 Gb for *A. atlantica* and 40 Gb for *A. eriantha* of 2 × 150 bp Illumina sequences were generated. A k-mer analysis (at *k* = 21 scale) using Genoscope [31] predicted a genome size of 3.72 Gb with 0.07% heterozygosity and a repeat fraction of 78% for *A. atlantica* and a genome size of 4.17 Gb with a 0.12% heterozygosity and a repeat fraction of 76% for *A. eriantha*. The relative magnitude of these values agree well with those reported by Bennett and Smith [26] and Yan et al. [32], both of which report that the genomes of the A-genome diploids are ~ 15% smaller than the C-genome diploids. However, former estimates determined by replicated flow cytometry measurements ranged in size from 4.1 to 4.6 Gb for A-genomes and from 5.0 to 5.1 Gb for C-genomes [32]. The differences in genome size predicted by k-mer vs. flow cytometry analyses are likely a reflection of the significant repeat fraction in the oat genome that is difficult to account for using a k-mer analysis.

Prior to Hi-C scaffolding, Canu was used to assemble the *A. atlantica* and *A. eriantha* PacBio long reads into 3914 and 8067 contigs with an N50 of 5,544,947 and 1,385,002 bp, spanning a total of 3.68 and 3.77 Gb of total length, respectively (Table 1). The L50 of the assemblies were 196 and 797 and the longest contigs spanned 25,143,700 and 10,103,775 bp, respectively. The average G+C content of the assemblies were 44.4% and 43.9%, which is similar to most monocotyledonous cereals (e.g., *Sorghum bicolor*, 43.9%; *Oryza sativa*, 43.6% G+C) but significantly higher than G+C content predicted for dicots, which typically range between 33 and 36% (e.g., *Carica papaya*, 34%; *Arabidopsis thaliana*, 36%) [34]. As these were PacBio read based assemblies, no “N” gaps were present in the Canu assemblies.

**Table 1:** Summary statistics for the canu [33] and Hi-C assemblies for *A. atlantica* and *A. eriantha*

Assembly	*A. atlantica*		*A. eriantha*	
	Canu	Hi-C	Canu	Hi-C
Number of scaffolds	3941	2195	8067	2652
Total size of scaffolds (bp)	3,683,522,149	3,685,054,491	3,773,539,112	3,777,787,481
Longest scaffold (bp)	25,143,700	577,845,554	10,103,775	588,203,704
Shortest scaffold (bp)	1010	1010	1020	1020
Number of scaffolds > 1 M nucleotides	768	9	1203	7
N50 scaffold length	5,544,947	513,237,590	1,385,002	534,821,622
L50 scaffold count	196	4	797	4
Scaffold % A	27.81	27.81	28.06	28.04
Scaffold % C	22.2	22.19	21.94	21.91
Scaffold % G	22.19	22.18	21.93	21.91
Scaffold % T	27.8	27.79	28.07	28.05
Scaffold % N	0	0.03	0	0.09
Scaffold N nt	0	1,250,201	0	3,223,400
Scaffold % non-ACGTN	0	0	0	0
Percentage of assembly in scaffolded contigs	0.00%	97.00%	0.00%	97.80%
Average number of contigs per scaffold	1	1.9	1	3.1
Average length of breaks (20 or more Ns) between contigs	0	601	0	578
Number of contigs	3941	4275	8067	8228
Number of contigs in scaffolds	0	2244	0	5740
Number of contigs not in scaffolds	3941	2031	8067	2488
Total size of contigs	3,683,522,149	3,683,804,291	3,773,539,112	3,774,564,081
Longest contig	25,143,700	21,736,085	10,103,775	10,106,525
Shortest contig	1010	120	1020	198
Number of contigs > 1 M nt	768	868	1203	1202
N50 contig length	5,544,947	4,310,367	1,385,002	1,314,218
L50 contig count	196	245	797	838
Contig % A	27.81	27.81	28.06	28.07
Contig % C	22.2	22.2	21.94	21.93
Contig % G	22.19	22.19	21.93	21.93
Contig % T	27.8	27.8	28.07	28.07
Contig % N	0	0	0	0
Contig %non-ACGTN	0	0	0	0

To improve the Canu assemblies, contigs were further scaffolded by chromatin-contact maps using DoveTail Chicago® and Hi-C libraries. Chicago® library contact maps are based on purified DNA that is reconstituted in vitro and thus limited to chromatin associations no larger than the size of the purified input DNA fragments (< 100 kb). Nonetheless, they are ideal for detecting and correcting miss-joins in de novo assemblies as well as short-range scaffolding [35]. Approximately 73× coverage of 1–100 kb read pairs (2 × 150) were generated from Chicago® libraries for each *Avena* species and used to scaffold the Canu assemblies using the HiRiSE™ scaffolder. In total, 334 and 158 breaks were made, while 1157 and 2962 joins were made in the *A. atlantica* and *A. eriantha* assemblies, respectively. The net effect of these changes was to decrease the number of total scaffolds to 3118 in the *A. atlantica* assembly and to 5263 in the *A. eriantha* assembly, which was accompanied by a slight decrease in N50 (4310 and 1.314 kb, respectively) for each assembly. Whenever a join was made between contigs, an “N” gap, consisting of 100 Ns, was created. The total percent of the genome in gaps, for both species, was less than 0.1%.

The Chicago®-based assemblies were then further scaffolded using in vivo Hi-C libraries, created from native chromatin to produce ultra-long-range mate pairs. Mate pair reads (10–10,000 kb) representing a physical coverage of 2749× and 513× were generated for the *A. atlantica* and *A. eriantha* genomes and scaffolded using the HiRiSE™ scaffolder. In total, 922 joins and 2614 joins (plus three breaks) in the *A. atlantica* and *A. eriantha* were made, respectively, producing ultra-long scaffolds, putatively representing full-length chromosomes and/or chromosome arms. The HiRiSE™ assembly for *A. atlantica* had a scaffold N50 of 513.2 Mb, and an L50 of 4, spanning a total sequence length of 3.685 Gb. The longest scaffold spanned 577.8 Mb. Similarly, the *A. eriantha* assembly had a scaffold N50 of 534.8 Mb, an L50 of 4, and spanned a total sequence length of 3.778 Gb with the longest scaffold reaching 588.3 Mb. Scaffold joins produced by the Hi-C mate pairs introduced new “N” gaps in the assembly (each consisting of 1000 Ns), thereby increasing the number of gaps in the assembly to 2079 and 5576 for *A. atlantica* and *A. eriantha*, respectively. The final percentage of “N” nucleotides in the final assemblies was less than 0.1%, with the average gap size of 600 and 578 bp, respectively (Table 1).

The longest eight scaffolds of the *A. atlantica* assembly, presumably representing two chromosome arms (205 and 278 Mb) and six full-length chromosomes (448–577 Mb), consisted of > 96% of the total sequence length from the Canu assembly. Similarly, the longest seven scaffolds, ranging in size from 455 to 588 Mb, presumably representing each of the seven haploid *A. eriantha* chromosomes, were composed of > 97% of the total Canu assembly sequence. For simplicity, scaffolds representing each of the seven chromosomes from each species are referred to forthwith by size (longest to shortest) as AA1-AA7 and AE1-AE7. The scaffolds in the *A. atlantica* and *A. eriantha* assemblies that remain unintegrated into one of the chromosome-scale pseudomolecules are relatively small and repetitive, with an average size of 61 and 38 kb, which likely contributed to the inability of the proximity-guided assembler to confidently place these contigs within the framework of the chromosomes—specifically the low number of interactions on a short fragment as well as the inability to discern interaction distance differences over the short molecule.

### Chromosome arm merging

**Fig. 1:**
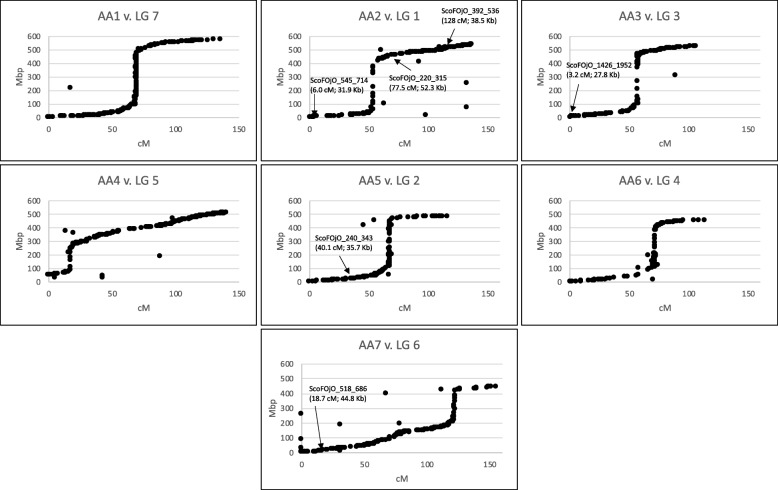
Correlation between the physical and linkage map. The genetic position of mapped markers is plotted as a function of physical distance relative to the *A. atlantica* genome assembly. The linkage position of six unassigned scaffolds with multiple mapping markers is shown

We compared the *A. atlantica* assembly with a recently published genetic linkage map, constructed from an F_6:8_ recombinant inbred line population generated from a cross of *A. strigosa* x *A. wiestii*, both A_s_A_s_ *Avena* diploid species [36]. This map was based on 11,455 ordered, codominant 64-base tag-level haplotypes on seven linkage groups generated using the Haplotag pipeline [37]. Of these, 4551 haplotypes had perfect matches to single sites on the eight largest scaffolds. A clear one-to-one correspondence between linkage groups (LG) and physical assembly scaffolds was observed (Fig. 1), with greater than 97% of the tag-level haplotypes mapping to a specific scaffold derived from a single LG. For example, of the 846 tag-level haplotypes mapping to scaffold ScoFOjO_324_449 (AA1), 838 (> 99%) were derived from LG 7 (Table 2). Of the 464 tag-level haplotypes derived from LG 2, 378 mapped to scaffold ScoFOjO_1310 (278 Mb) and 85 mapped to scaffold ScoFOjO_1577 (205 Mb), indicating that these two smaller scaffolds should be merged to produce a single, full-length pseudo chromosome (AA5; 485 Mb), thus completing the assembly of seven full-length haploid chromosomes for *A. atlantica*. A head-to-tail merging of these chromosome arms (separated by 1000 Ns) was determined based on the collinearity of the tag-level haplotypes with respect to their orientation within the linkage group. A near perfect collinear relationship was observed between the linkage map and the physical map for all chromosome-linkage group comparisons, with the exceptions being the anticipated reductions of linkage distances relative to physical distances observed at the pericentromeric regions of each chromosome (Fig. 1). It is well documented that recombination is suppressed in centromeres at rates ranging from fivefold to greater than 200-fold, depending on the species [38, 39]. Of the 2188 contigs that were unintegrated into an *A. atlantica* chromosome using the Hi-C data, we identified segregating haplotypes linked to 22, spanning a total length of 1.07 Mb, which could tentatively place them into the context of the seven haploid chromosomes based on their linkage position (Fig. 1).

**Table 2:** Physical map and Linkage map assignment. Haplotag markers from the consensus map of Latta et al. [36] where used to assign scaffold assemblies to linkage groups. Two scaffolds mapped to LG 2 and were merged

LG	Total markers	Miss-matches	% Miss-matches	% Matches	*A. atlantica Hi-C* scaffold	*A. atlantica* chromosome
1	705	12	1.7%	98.3%	ScoFOjO_1702_2338	AA2
2A^1^	85	2	2.4%	97.6%	ScoFOjO_1577	AA5
2B^1^	378	10	2.6%	97.4%	ScoFOjO_1310	
3	546	11	2.0%	98.0%	ScoFOjO_2069_2732	AA3
4	370	16	4.3%	95.7%	ScoFOjO_2050_2712	AA6
5	872	24	2.8%	97.2%	ScoFOjO_350_483	AA4
6	749	36	4.8%	95.2%	ScoFOjO_1760_2399	AA7
7	846	8	0.9%	99.1%	ScoFOjO_324_449	AA1
Total:	4551	119	2.7%	97.3%	–	–

^1^A = Markers spans 205.8 on the physical map corresponding to linkage positions 0–48 cM on the consensus linkage map; B = Markers spans 278.2 Mb on the physical map corresponding to linkage position 49–116 cM on the consensus linkage map

### Analysis of repetitive elements

The repeat fraction of the *Avena* genome assemblies was identified and annotated using RepeatModeler and RepeatMasker. In total, ~ 83% of each genome was classified as repetitive, with the most commonly identified repetitive elements being classified as long terminal repeat retrotransposons (LTR-RTs); LTR-RTs are the most abundant genomic components in flowering plants [40, 41], and their abundance is strongly correlated with genome size [42]. Within published plant genomes, repeat content varies widely, ranging from 3% for the minute 82 Mb genome of *Utricularia gibba* L. [43] to 85% for maize [44]. Given the large size of these genomes, it is not surprising that < 20% of the genome is classified as non-repetitive.

**Fig. 2:**
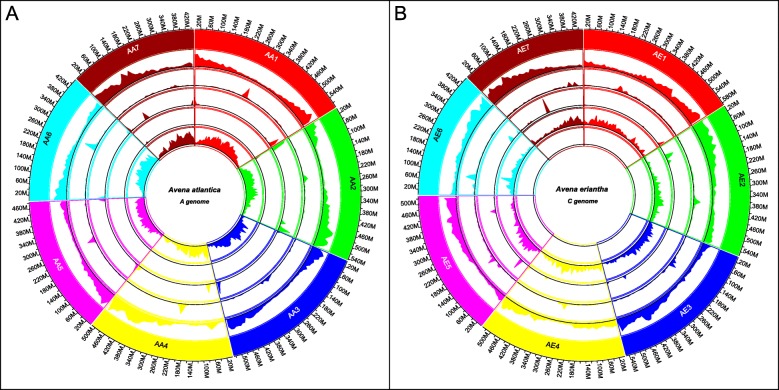
Genome overview of **a** *A. atlantica* and **b** *A. eriantha*. Track 1 (outside): Chromosome and sizes; Tracks 2: Annotated gene density; Track 3: Centromeric repeat density; Track 4: Telomeric sub-repeat density; Track 5: C-genome specific repeat (pAm1) density; Track 6: A-genome specific repeat (pAvKB26) density

Of the various LTR-RT present (Additional file 1: Table S1), *Gypsy*-like and *Copia*-like elements represent > 60% of each genome, in a ratio of 2.3:1 and 3.5:1 for the *A. atlantica* and *A. eriantha* genomes, respectively, which is similar to the ratios reported for other Poaceae species (e.g., rice, 4.9:1 [45]; sorghum, 3.7:1 [46]; and maize, 1.6:1, [47]). The next most common element was class II CMC-EnSpm DNA transposons, representing ~ 5% of each genome—which are known common features of the cereals [48, 49]. Interestingly, a significant proportion (*A. atlantica*: 10.6% and *A. eriantha*: 14.3%) of the interspersed repeat fraction for each genome was classified as “unknown”. Given the extensive investigations of repeat elements in the grasses [50–52], this unknown fraction likely represents repeat elements unique to *Avena* and could be invaluable in differentiating the A-, C-, and D-subgenomes in hexaploid oat. For example, Solano et al. [53] reported the identification of a tandem repeat sequence clone (pAm1; GenBank X83958) from *Avena murphyi* L., an AACC tetraploid, which selectively hybridized to the C-subgenome. A repeat that was highly homologous (E-value 2E-82) to pAm1 was identified by RepeatModeler in *A. eriantha*, but is missing in the *A. atlantica* genome (Fig. 2a; Tracks 4 and 5). Similarly, Katsiotis et al. [54] reported the identification of an interspersed repeat (pAvKB26; GenBank AJ297385.1) that selectively hybridized to only the A- and D-subgenomes. This repeat was identified in the unknown repeat fraction of *A. atlantica* but was missing in the *A. eriantha* genome (Fig. 2b; Tracks 4 and 5). Repeat content is believed to be an important driver of genome organization and evolution [55] and these data will be important for understanding the overall evolution of common hexaploid oat.

In addition to the interspersed repeat elements, ~ 0.5% of the genome was classified as low complexity, satellite, microsatellite or telomeric repeat (see genomic feature section below). Indeed, 5217 and 3404 putative microsatellite loci were identified, with the most common di-, tri- and tetranucleotide repeat motif identified being (AT)_n_, (AAC)_n_ or (GGC)_n_ and (TTTA)_n_, in *A. atlantica* and *A. eriantha*, respectively. To date, no microsatellites have been generated specifically for the *Avena* diploid species – thus these new putative microsatellite loci represent important genetic tools for studying diversity and specifically for advancing breeding in the A-genome diploids.

### Transcriptome assembly and functional annotation

The *A. atlantica* and *A. eriantha* transcriptomes, which consisted of 51,223 and 47,361 scaffolded isoforms, the *Brachypodium* cDNA and peptide models (v 1.0; Ensembl genomes) and the uniprot-sprot database were provided as primary evidence for annotation in the MAKER pipeline [56]. The RNA-Seq data mapped with high efficiency to the assemblies, with > 96% of the reads mapping to their respective genome at 93.1% concordance for pair alignment rates, suggestive of high-quality genome assemblies for both species. The MAKER pipeline identified a total of 51,100 and 49,105 gene predictions, with mean transcript lengths of 3018 and 3153 bp, and with 70% and 66% of the annotations having annotation edit distance (AED) measures < 0.25, for *A. atlantica* and *A. eriantha* genomes, respectively. AED integrates sensitivity, specificity, and accuracy measurement to calculate annotation quality, where AED values < 0.25 are indicative of high-quality annotations [57]. The mean G+C content of the transcripts in both species was ~ 52%. The increase in G+C content within coding regions relative to the overall G+C content of the genome (~ 44%) is a well-known phenomenon and is hypothesized to be the result of GC-biased gene conversion – a process by which the G+C content of DNA increases due to gene conversion during recombination [58].

The completeness of the gene space was quantified using BUSCO which provides a quantitative measure for genome and transcriptome completeness based on a core set of highly conserved plant-specific single-copy orthologs [59]. Of the 1440 plant-specific orthologs, 1387 (96.3%) were identified in the *A. atlantica* genome assembly as full length, while 1395 (96.9%) were identified in the *A. eriantha* assembly as full length, suggesting high-quality and complete genome assemblies. As expected for diploid species, the level of gene duplication, as identified by BUSCO for the conserved orthologous genes was low for both species (2.2% and 2.3%). Similarly, a BUSCO analysis of the transcript and protein annotation sets produced by MAKER identified similar numbers of conserved orthologs for both species, which is indicative of a successful annotation process.

### Genomic features

Pericentromeric regions, associated with reduced recombination relative to physical distance, were evident from the linkage and physical map comparison in *A. atlantica* (Fig. 1). The observation that gene density is substantially reduced provided further evidence that that these regions represented centromeric regions in both species, as has been well-documented previously in other eukaryotes [60, 61]. Centromeres in most plant species are complex but are dominated by megabase-sized arrays of tandemly arranged monomeric satellite repeats. While complex and highly diverse among plant species, they commonly share a unit length ranging between 150 and 180 bp, which is close to the size of the nucleosome unit [62]. Melters et al. [63] showed that due to the relative size of the centromere, the most common repeat found in whole-genome sequencing data is the putative centromeric repeat. Using the output of RepeatModeler from *A. eriantha*, we identified a high-copy-number 159 bp tandem repeat that aligned specifically with the putative centromere location in each of the *A. atlantica* and *A. eriantha* chromosomes (Fig. 2; Additional file 2). Although the 159 bp repeat is similar in size to the putative centromeric repeats found in other grass species (e.g., *B. distachyon*, 156 bp; *H. vulgare,* 139 bp; *Oryza brachyantha* A.Chev. & Roehr.*,* 154 bp; *Z. mays*, 156 bp), not surprisingly it shares little sequence similarity with the centromeric repeats of those species. Indeed, centromeric repeats exhibit little to no evidence of sequence similarity beyond ~ 50 million years of divergence [63]. As has been documented in other plant species, these putative centromeric repeats span a large portion (often > 50 Mb) of the *A. atlantica* and *A. eriantha* chromosomes, suggesting the presence of large pericentromeric heterochromatic regions [64, 65]. Moreover, the positioning of the centromeres, as defined by this putative repeat and the gene density plots, is consistent with the cytological positioning of the centromere, which suggests that the centromeres in *A. atlantica* are almost all metacentric to submetacentric, while the centromeres in *A. eriantha* are almost all sub-telocentric [66, 67]. Indeed, per our analyses, we identified three metacentric, two submetacentric and two sub-telocentric chromosomes in *A. atlantica*, and five sub-telocentric, one submetacentric and one metacentric chromosome in *A. eriantha* (Fig. 2).

Repeatmodeler annotated a putative telomeric satellite sequence (665 bp) for *A. eriantha* (Additional file 1: Table S1). A homologous sequence (639 bp) with significant homology (E-value = 0.0) and alignment identity (Identity = 80%; Gap = 6%) was identified from the repeat sequences identified by RepeatModeler (Additional file 2). BLAST searches of the assemblies with their respective satellite telomeric repeat sequence identified enriched regions of the telomeric repeat on all seven of the chromosomes for each species (Fig. 2). In *A. atlantica*, telomeric satellite sequences were located toward the distal end of each chromosome; however, in *A. eriantha* the location of the sequence is more dispersed, being found primarily at the end of the chromosomes on ten of the 14 chromosome arms, while in a few instances being interspersed interstitially. Interstitial telomere-like repeats have been reported in several plant species including *Anthurium*, *Vicia*, *Sideritis*, *Typhonium,* and *Pinus* where they were implicated in chromosome rearrangements, including inversions, translocations, and chromosome fusions [68–71]. While chromosomal rearrangements are common in *Avena*, we caution that the very repetitive nature of telomeric sequences makes them susceptible to collapse during the assembly process and are thus inherently difficult to order and orient in the Hi-C scaffolding process.

### SNP discovery and genetic diversity

To characterize the diversity and phylogenetic relationships among *Avena* A- and C-genome diploid species, we resequenced at 10X coverage 61 A-genome diploid accessions (including *A. atlantica, A. brevis, A. canariensis, A. damascena, A. hirtula, A. longiglumis, A. lusitanica, A. strigosa, A. strigosa-brevis, A. strigosa-hispanica, A. strigosa-nuda,* and *A. wiestii*) and 10 C-genome diploids (*A. clauda, A. eriantha, A. ventricosa*; Additional file 3: Table S2). The resequencing produced 40 Gb sequence data per accession (Additional file 3: Table S2). The A-genome accession reads were mapped against the *A. atlantica* genome, while the C-genome species were mapped against the *A. eriantha* genome for SNP discovery using InterSnp [72]. InterSnp uses BAM files to call SNPs between samples based on consensus alleles at each genomic position, filtered to produce a dataset with 0% missing data across all lines. Considering the cleistogamous nature of the accessions included, any SNPs with > 5% heterozygous calls were deemed likely to result from spurious read-mapping and were removed from the dataset. Using a minimum allele frequency threshold of < 0.1, a total of 286,567 and 3,185,959 putative SNPs were identified within the A-genome and C-genome diploid datasets, respectively, and used by SNPhylo [73] to investigate phylogenetic relationships. SNPhylo reduces oversampling effects at linked SNPs using an LD threshold (0.1) with a sliding window of 500,000 base pairs. Thus, a total of 7221 and 11,530 SNPs, with an average of 1032 and 1647 SNPs per chromosome, were selected prior to tree construction for the A-genome and C-genome diploids dataset, respectively (Additional file 4: Table S3).

The bootstrapped maximum likelihood phylogenetic trees were rooted with either the *A. eriantha* accession CN 19328 for the A-genome accessions tree (Fig. 5a) or with the *A. atlantica* accession Cc 7277 for the C-genome accessions tree (Fig. 5b). The A-genome diploids formed two distinct clades: one of these consisted primarily of accessions classified in taxa having the A_s_A_s_ subgenome, which had previously been described by Rajhathy and Morrison [74] and Leggett [75] as including *A. atlantica*, *A. hirtula*, the domesticated forms of *A. strigosa*, and *A. wiestii*; and a second clade comprised mostly of *A. canariensis* (A_c_A_c_), Syrian accessions of *A. damascena* (A_d_A_d_), *A. longiglumis* (A_l_A_l_), and three floret-shattering accessions that were possibly misidentified as *A. hirtula* and *A. lusitanica*. As expected, the spikelet-shattering *A. atlantica* occupied the basal position on the A_s_A_s_ branch of the tree, while all of the *A. strigosa* (domesticated A_s_A_s_) genotypes formed a clade at the top of the tree and included a single accession of weedy *A. wiestii* (CIav 1994) that, upon inspection of the panicles, more closely resembled a long-awned, semi-shattering *A. strigosa* genotype.

The *A. strigosa* branch shows clearly the effect of a domestication bottleneck. This branch of the tree is subdivided into two distinct sub-branches. The upper sub-branch consists predominantly of genotypes of Iberian origin (i.e., CN 25698, CIav 9019, CIav 9036, etc.) and includes seven homogeneous accessions that are derivatives of the Brazilian ‘Saia’ variety of forage oat (i.e., CIav 7010, PI 291990, etc.). Interestingly, the *A. hispanica* genotypes form a unitary subclade within this branch that is strongly supported by the bootstrap value. The lower sub-branch is comprised of accessions from outside the Iberian Peninsula (PI 83721, PI 287314, PI 304557, CIav 9022, etc.) and includes all of the *A. strigosa-nuda* varieties. Since *A. brevis* strains are distributed in both branches, there is no evidence to confirm its identity as a distinct taxon within or apart from *A. strigosa*. The presence of branches containing multiple, genetically indistinct accessions indicates there is a high degree of duplication being curated within the USDA and PGR-Canada gene banks for *A. strigosa*.

The remainder of the A-genome tree consists of entirely wild genotypes. *Avena lusitanica* is not a universally accepted taxon, and the presence of these strains on various branches of the tree confirms that this is not a valid independent taxonomic entity; instead, it is part of the floret-dispersing *A. hirtula-wiestii* complex of semi-desert and Mediterranean scrub ecotypes of the A_s_A_s_ biological species complex. The presence of three floret-shattering accessions from Morocco that were previously identified as *A. damascena* (A_d_A_d_) on the A_s_A_s_ branch (PI 657468, PI 657471, PI 657472) and the two Syrian *A. damascena* genotypes on the other branch (CN 19457, CN 19459) suggests that the Moroccan group are misidentified and are therefore, like *A. lusitanica*, either members of the A_s_A_s_ *A. hirtula-wiestii-atlantica-strigosa* complex or, possibly, misclassified accessions of tetraploid *A. barbata*.

The rooted C-genome tree had the lone *A. ventricosa* (C_v_C_v_) accession at the base of the C-genome branch that consisted of two subclades. The more basal branch consisted of accessions of spikelet-shattering *A. eriantha* (C_p_C_p_) from Algeria (CN 24022) and four samples of *A. eriantha* from an extended population growing in the Middle Atlas Mountains of Morocco (PI 657575–8). The other branch included Algerian (CN 24040) and Turkish (CN 19238) accessions of floret-shattering *A. clauda* (C_p_C_p_) along with Iranian (CN 19256) and Algerian (CN 19328, the reference genome) *A. eriantha* genotypes.

## Discussion

### Comparative genomics

The age of the Poaceae family has been difficult to establish, with varying ages reported in the literature [76, 77]. Schubert et al. [78] recently reported the use of newly available paleobotanical fossils to established the age of the family to be approximately 120.8 million years ago (Ma), with the split of the Aveneae, Brachypodieae, and BOP clades occurring approximately 44.3, 51.8, and 80.2 Ma, respectively - suggesting that the grasses have a lower nucleotide substitution rate than the other angiosperms [79]. We calculated the rate of synonymous nucleotide substitutions per synonymous site (K_s_) for orthologous gene pairs between the *A. atlantica* and *A. eriantha* assemblies using the CodeML [80] tool on the CoGe platform (genomevolution.org/coge). A total of 18,002 duplicate gene pairs were identified with a clear peak seen at K_s_ = 0.0875 (Additional file 5: Figure S1). From the node estimates reported by Schubert et al., we calculated an average substitution rate for the Pooideae lineage of 3.39E-09, suggesting that speciation between *A. atlantica* and *A. eriantha* occurred between 5.4–12.9 million years ago (Ma), depending on whether a core eukaryotic-based synonymous mutation rate or the calculated lineage specific rate for Pooideae was used in the calculation [81, 82]. As seen in the SynMap dotplot alignment (Additional file 6: Figure S2), significant synteny was observed between the *Avena* chromosomes consisting of 187 syntenic blocks with 21,021 collinear genes pairs (112 genes/block) with 98.2% coverage across both the *A. atlantica* and *A. eriantha* genomes. As expected, given the relatively close ancestry of the species, the size (bp) of the syntenic blocks between species was highly correlated (R^2^ = 0.88; Additional file 6: Figure S2C). The large blocks of syntenic genes are suggestive of orthologous relationships between the chromosomes of the species (Additional file 6: Figure S2A). For example, slightly more than 77% (349 Mb) of the syntenic sequence found on AA2 is derived from AE5, suggesting that they are orthologs. Indeed, using a majority rule (> 50% syntenic sequence) we identified the following orthologous chromosome pairs: AA1 + AE6 (61%; 248 Mb); AA2 + AE5 (77%; 349 Mb); AA3 + AE3 (74%; 318 Mb); AA4 + AA1 (71%; 271 Mb); AA7 + AE2 (57%; 274 Mb); with AA5 and AA6 sharing orthology with several *A. eriantha* chromosomes (Additional file 6: Figure S2B).

The Poaceae family consists of many agronomically important species, commonly referred to as cereals, that are found in three subfamilies: Oryzoideae (rice), Panicoideae (maize, sorghum) and Pooideae (wheat, barley, oat and rye). Pooideae forms 14 tribes, including the tribes Brachypodieae, Poeae (syn Aveneae, including oat) and Triticeae (barley, rye, and wheat), with Poeae and Triticeae tribes having separated ~ 49 Ma [78]. This agrees well with the K_s_ analyses presented here for *A. atlantica* and *A. eriantha* and with the published *Hordeum vulgare* genome [83], which both show a clear peak at 0.3 – suggestive of a divergence time of 44 Ma (per the calculated lineage specific rate for Pooideae). As expected, the K_s_ analyses from the *Avena* comparisons with the *B. distachyon* genome (International *Brachypodium* Initiative, 2010) suggested a more distant divergence of 47–51 Ma for the split of the *Avena*–*Brachypodium* lineages (Additional file 5: Figure S1).

SynMap was also used to investigate syntenic relationships between the *Avena* and *Hordeum* chromosomes (Additional file 7: Figure S3 and Additional file 8: Figure S4). Although more syntenic blocks (719 and 714) were identified in the *Avena–Hordeum* comparisons, they were smaller – consisting of ~ 8.5 genes/block, accompanied by a lower syntenic block size correlation (R^2^ = 0.35 and 0.41; Additional file 7: Figure S3C and Additional file 8: Figure S4C). The decrease in block size and correlation is reflective of the more distant evolutionary relationship between the species. Nonetheless, the shared ancestry between the two Pooideae species was evident as seen by substantial synteny observed across all seven *Avena–Hordeum* chromosomes comparisons (Additional file 7: Figure S3A and Additional file 8: Figure S4A). As expected, large, proximal, non-syntenic regions were observed in regions corresponding to putative centromeres where gene density is substantially reduced [60, 61]. The synteny observed among the *Avena* and *Hordeum* chromosomes suggests several homeologous relationships. For example, *Hordeum* chromosome 1H is clearly orthologous with *Avena* chromosome AA2 and AE5. Indeed, of the syntenic sequence on 1H, 99% (116 Mb) was syntenic to AA2 and 85% (105 Mb) syntenic to AE5 – which is not surprising since we previously showed that AA2 and AE5 are orthologs (see above). Using a simple majority rule (> 50% syntenic sequence) the following are putative *Hordeum*–*Avena* orthologs: 1H + AA2/AE5; 2H + AA5/AE4; 3H + AA3/AE3; 6H + AA7**/**AE2; and 7H + AA1/AE6. The specific *A. atlantica* orthologs of 4H and 5H are likely AA6 and AA4, respectively; however, rearrangements obscure the likely orthologs for *A. eriantha* (Additional file 7: Figure S3B and Additional file 8: Figure S4B).

Bekele et al. [29] recently published a high-density, tag-level haplotype linkage map of hexaploid oat (*A. sativa*). This consensus linkage map increased the marker density of the former consensus map [28] consisting of 21 well-formed linkage groups, putatively corresponding to each of the 21 hexaploid oat chromosomes. To identify the ancestral subgenome groups (A-, C- and D-) for each of the 21 linkages groups we mapped the haplotag markers to both the *A. atlantica* and *A. eriantha* genomes. To avoid false hits, which is particularly problematic due to the highly repetitive nature of the oat genomes, only BLAST hits with perfect identity across the entirety of the marker sequence (e.g., zero gap openings and mismatches) were retained for downstream analyses. In total, 2119 and 969 tag-level haplotypes were mapped to the *A. atlantica* and *A. eriantha* genomes, respectively. The increased number (~2X) of tag-level haplotypes mapping successfully against the *A. atlantica* genome was expected since many D-subgenome haplotypes would map against the A-genome diploid, given the close phylogenetic relationship of these two subgenomes [16]. Indeed, close inspection of the mapping showed that in nearly all cases, tag-level haplotypes mapping to a specific *A. atlantica* chromosome were derived from two separate consensus linkage groups - presumably corresponding to homoeologs derived from A- and D-subgenomes. For example, 322 tag-level haplotypes mapped to chromosome AA1, with 153 (48%) derived from linkage group Mrg12 and 111 (35%) derived from linkage group Mrg02, which were previously identified as being derived from the A-, and D- subgenomes [16] (Table 3A). Other homoeologous chromosome pairs between the A- and D-subgenome included: Mrg33/Mrg08, Mrg18/Mrg01, Mrg05/Mrg04, Mrg24/Mrg06, Mrg23/Mrg11, Mrg12/Mrg02, and Mrg20/Mrg21. Similar mapping of the tag-level haplotypes against the *A. eriantha* genome elucidated linkage groups Mrg13, Mrg03, Mrg15, Mrg17, Mrg19, Mrg09 and Mrg 11 as being derived from C-subgenome (Table 3B). Interestingly, Mrg18, which we previously designated as an A-subgenome derived linkage group also showed substantial mapping to the C-genome chromosome AE7 – suggesting that this Mrg18 is actually derived from an A-subgenome/C- subgenome (A/C) intergenomic reciprocal translocation. This is a well-documented reciprocal translocation, first reported by Jellen et al. [84] where it was identified as 7C-17A. Other identifiable rearrangements include D- subgenome and C-subgenome (D/C) intergenomic exchanges on Mrg06/Mrg13, Mrg08/Mrg03, and Mrg19/Mrg28 (Table 3).

**Table 3:**
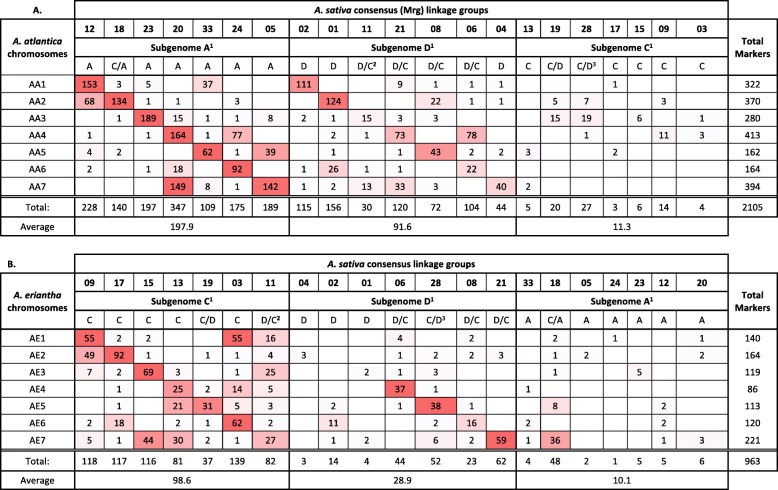
Ancestral subgenome groups (A-, C- and D-) designation for each of the 21 consensus linkages groups reported for *A. sativa* [29]. Haplotag markers mapping to (A) *A. atlantica* and (B) *A. eriantha* chromosomes, where highest haplotag mapping are colored red and transition to white as the number of haplotags mapping decreases

^1^Chromosomal designation as previously reported by Chaffin et al. [28] and Yan et al. [16]. Where assignments are split by a forward slash, the assignment given to the longest part of the chromosome is shown first

^**2**^Chaffin designated Mrg11 as C/A, while Yan et al. designated it as C. Here we assign it as D/C

^3^Yan et al. [16] designated Mrg28 as D/C, while Chaffin et al. [28] designated it as C. Here we re-designate it as D/C

### Utility of the genome assemblies

**Fig. 3:**
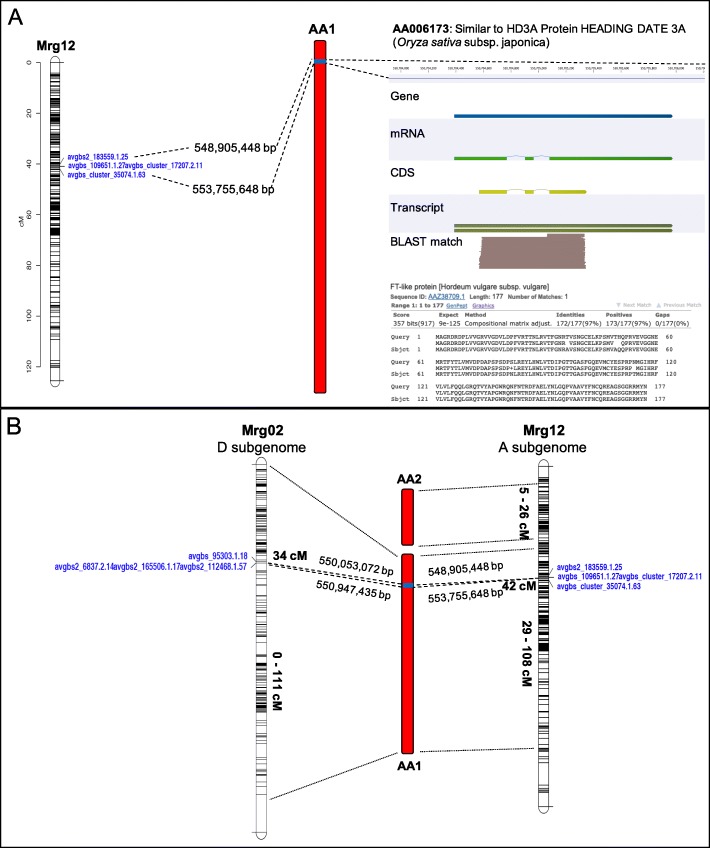
Identification of candidate genes putatively underlying heading date in oats. Candidate gene loci were identified using BLAST searches against the *A. atlantica* genome assembly using maker sequences associated with heading date QTLs located on the homoeologous linkage groups **a** Mrg12 and **b** Mrg02 (Bekele et al. [29]). Markers from both QTLs mapped to the same physical position on chromosome AA1, within an interval containing an FT-like protein (HD3A), suggesting that heading date in modern oat is controlled by two functional homoeologs of the flowering time gene

Given the genetic complexity of polyploid species, diploid species have frequently been used as simplified genetic models [85–87]. We show the value of these diploid assemblies using published genome wide association studies (GWAS) for heading date and crown rust resistance - both major breeding targets for common oat. Heading date (flowering time) is critically important for regional adaptation, photosynthetic efficiency, and stress avoidance; and through these factors it strongly influences overall yield [88]. A Haplotag-based GWAS of heading date in the CORE diversity panel (*n* = 635) of common hexaploid (AACCDD) oat identified two major associations on linkage groups Mrg02, at position 34 cM in eight field trails and on Mrg12 at position 40–42 cM in seven field trials [29]. Interestingly, our comparative analysis (see above) suggests that Mrg02 and Mrg12 are homoeologous (Table 3A), with Mrg12 and Mrg02 being derived from the A-subgenome and D-subgenome, respectively. BLAST searches against the *A. atlantica* genome assembly using the maker sequences associated with heading date on Mrg12 localized the heading date quantitative trail loci (QTL) to chromosome AA1, at an interval spanning bases 548,905,448 – 553,755,648. A total of 175 annotated gene sequence are found within this region, including a likely candidate gene at the center of this interval, AA006173 (Fig. 3a; 550,704,569–555,704,964), which is annotated as being homologous to HD3A (Heading Date 3A) from *O. sativa*, and is homologous (E-value = 9e-125, Identity = 97%) to the flowering time protein (FT-like; AAZ38709.1). Yan et al. [89] described HD3A as the vernalization gene, VRN3, in wheat and barley. Interestingly, while Mrg02 is likely of a D-subgenome origin, BLAST search of markers associated with heading date from the Mrg02 linkage mapped significantly to the A-genome chromosome AA1 at an interval spanning 550,053,072–550,947,435 bp, only 242,471 bp from the aforementioned HD3A gene, suggesting that the candidate gene for both major QTLs for flowering time are functional homoeologs of the flowering time (FT) HD3A gene in the A- and D-subgenomes (Fig. 3b).

**Fig. 4:**
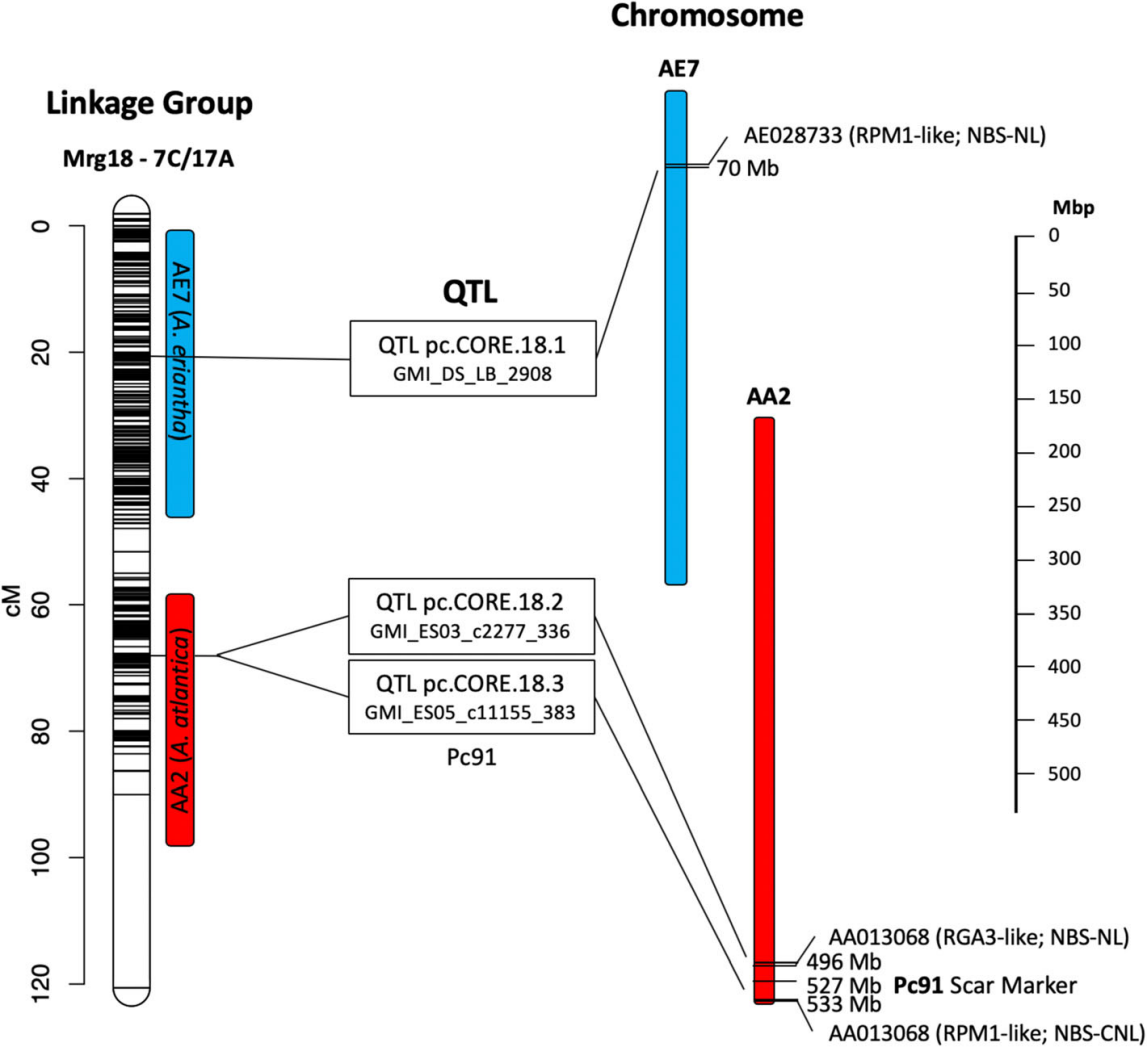
Identification of candidate genes putatively underlying crown rust resistance in oats. Candidate gene loci were identified using BLAST searches against the *A. atlantica* genome assembly using maker sequences associated with crown rust QTLs located on hexaploid *A. sativa* linkage group Mrg18 reported by Klos et al. [30]. Mrg18 was previously shown to be involved with an intergenomic translocation involving 7C and 17A, corresponding to *A. eriantha* chromosome AE7 (blue) and *A. atlantica* chromosome AA2 (red)

Crown rust, caused by *Puccinia coronata f. Sp. avenae*, is the most damaging and widespread disease of oat worldwide [90]. Moderate to severe outbreaks can reduce yield by 10–40% [91]. Klos et al. [30] performed a GWAS of crown rust resistance on elite common oat accessions challenged with multiple *P. coronata* isolates and identified multiple QTL on 12 linkage groups that were associated with crown rust resistance, several of which were associated with known resistance genes (e.g., *Pc91*). Resistance gene analogs (RGAs) contain specific conserved domains and motifs that can be used to identify and classify R-genes into four main RGA families: specifically, NBS-encoding proteins, receptor-like protein kinases (RLKs), receptor-like proteins (RLPs), and transmembrane coiled-coil proteins (TMCC). The RGAugury pipeline [92] annotated a total of 1563 (511 NBS, 722 RLK; 120 RLP; 160 TMCC) and 1402 (459 NBS; 654 RLK; 135 RLP; 154 TMCC) RGAs in the *A. atlantica* and *A. eriantha* genomes, respectively (Additional file 9: Table S4; Additional file 10: Figure S5). As has been observed in other monocots, no Toll/Interleukin-1 receptor-NBS-LRR R-genes were predicted in either genome, supporting the hypothesis that this class of R-gene evolved in eudicot lineage [93] or were lost during the evolution of the monocots [94]. The RGAs, specifically the NBS-encoding RGAs, cluster primarily in subtelomeric regions (Additional file 10: Figure S5), with clusters identified on almost all chromosomes and often correlated with the mapping position of crown rust QTLs. For example the *Pc91* gene, a known seedling resistance gene previously associated with QTL QPc.CORE.18.3 [30] maps, via two SNPs (GMI_ES03_c2277_336 and GMI_ES05_c11155_383), to the *A. atlantica* chromosome AA2 at positions 510,519,361 and 533,475,317, co-locating with a predicted disease gene cluster (Fig. 4). The closest annotated disease resistance genes to these markers are AA013376, similar to RPH8A (a nonfunctional homolog of rpp8 in Arabidopsis [95]) located at position 510,828,316 and AA014151, similar to RPM1 (a well-documented resistance gene in *Arabidopsis* [96]) located at 533,698,614 (Additional file 11: Table S5). Both candidate RGAs were identified by the RGAugury pipeline as CC-NBS-LRR containing R-genes. We caution that while these two candidate RGAs are positioned in the immediate vicinity of the associated markers, at least 87 RGA are present at the RGA cluster defined by the QTL. We note that the diagnostic SCAR and DART markers developed for *Pc91* also map to this same location (527,126,948 [97, 98];).

## Conclusions

Reference-quality, de novo whole-genome sequence assemblies for two highly repetitive ~ 4 Gb *Avena* diploid species were produced using a hybrid approach involving PacBio long reads, Illumina short reads, and both in vitro and in vivo chromatin-contact mapping. The whole-genome reference assemblies for A_s_- and C_p_-genome oat diploids provided for the first time in this paper represent powerful tools for identifying genes that underlie adaptive, disease resistance, and grain-quality traits critical for oat improvement. The utility of these whole-genome references was demonstrated first by analyzing sequences homologous to heading-date QTL-containing regions that were previously identified via GWAS in common hexaploid oat (*A. sativa*) to find linked candidate genes in *A. atlantica* and *A. eriantha*. Additionally, we used these references in successfully identifying RGAs homologous to oat crown rust resistance genes.

*Avena atlantica* retains a remarkable degree of synteny in comparison with barley while *A. eriantha* has undergone a relatively greater degree of chromosomal rearrangement, suggesting the presence of an underlying genomic instability in the C-genome diploids. This might be related to the abundant heterochromatin, including the underlying pAm1 repeat motif, distributed throughout the chromosomes of this genome (Fig. 2b, Track 5 [27];). Their genome sequences shed enormous insight into the complex evolutionary processes that have led to the appearance of cultivated diploid, tetraploid, and hexaploid oat going back millions of years. These processes included responses to natural selective events such as the Zanclean Cataclysm ~ 5 Ma and repeated cycles of global climate change characterized by boreal glacial maxima interspersed with humid periods and desertification due to northerly expansions of the Saharan and Arabian Deserts [99–101].

**Fig. 5:**
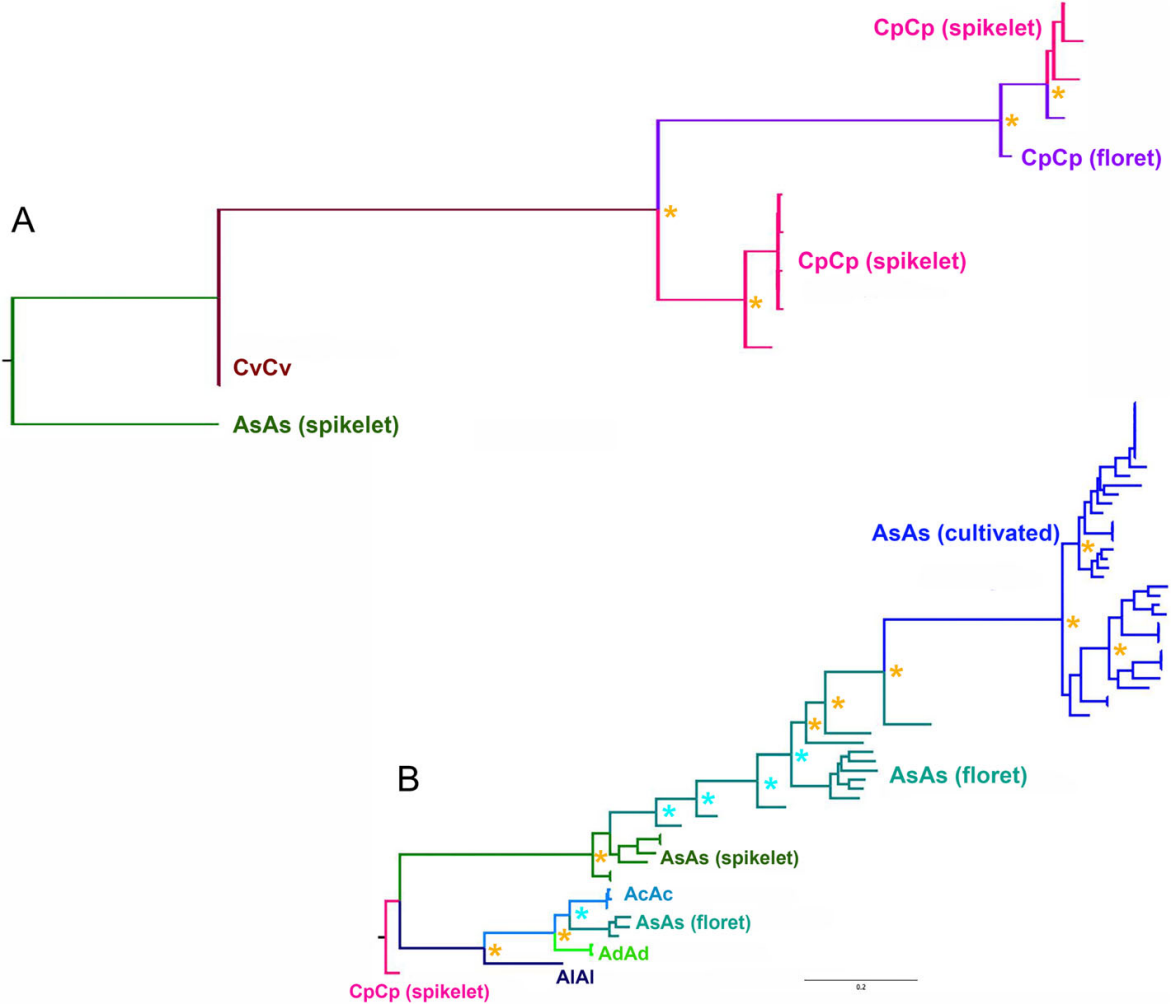
Abbreviated maximum likelihood tree generated using **a** 10,894 SNPs for C-genome diploids rooted to the *A. atlantica* (AT_Cc7277) reference and **b** 7221 SNPs for A-genome diploids rooted to the *A. eriantha* reference (ER_CN 19238). Asterisks denote percentage of 1000 bootstrap replicates that support the topology at 90–100% (gold) and 75–89% (blue). Scale bar represents substitutions per site. Branch labels are based on subgenome composition and, in some cases, diaspore morphology (“floret-shattering,” “spikelet-shattering,” or “cultivated”). Unabbreviated trees are provided as Additional file 15: Figure S7 and Additional file 16: Figure S8

We demonstrate that *A. atlantica*, *A. strigosa*, and *A. wiestii* represent multiple ecotypes or subspecies of a single biological species complex sharing the subgenome designation A_s_A_s_ and distinguished primarily by their seed-dispersal strategies. The phylogeny presented here, which was generated by analyzing thousands of SNPs identified via resequencing of dozens of geographically diverse accessions, clearly illustrates a monophyletic relationship with *A. atlantica* accessions at the root of the AsAs-genome clade (Fig. 5a). This is further seen in the high degree of synteny and collinearity between the *A. atlantica* chromosomes and *A. strigosa* X *wiestii* linkage groups reported by Kremer et al. [102] (Additional file 12: Figure S6). This result is remarkable, given the high degree of chromosomal rearrangement previously observed among different species and genomes within *Avena* [27].

The oat community has struggled without a reference genome for decades. Finally, we have complete references for what are, essentially, all three component genomes of cultivated hexaploid oat and the four known subgenomes of the genus, given the close correspondence between *Avena* subgenomes A, B, and D. Moreover, once a complete hexaploid reference is available, the utility of these component genomes will increase further, as they will provide a precise roadmap of the structural and functional evolutionary steps that took place in the formation of this unique and important polyploid species.

## Methods

### Plant material and nucleic acid extraction

For whole-genome assembly, young leaf tissue (~ 14–21 days post emergence), dark treated for 72 h, from *A. atlantica* (CC7277; T. Langdon, Aberystwyth University, Wales, UK) and *A. eriantha* (BYU132; EN Jellen, Brigham Young University, Provo, UT) was flash-frozen and sent to the Arizona Genomics Institute (AGI; Tucson, AZ, USA) for high molecular weight DNA extraction. For the diversity panel, DNA from 76 accessions of diploid A- and C-subgenome species (Additional file 3: Table S2) was extracted from 30 mg of freeze-dried leaf tissue using a protocol devised by Sambrook et al. [103] with modifications described by Todd and Vodkin [104]. All plants were grown in greenhouses at Brigham Young University (BYU) using Sunshine Mix II (Sun Gro, Bellevue, WA, USA) supplemented with Osmocote fertilizers (Scotts, Marysville, OH, USA) and maintained at 25 °C under broad-spectrum halogen lamps, with 12-h photoperiods.

### DNA sequencing and read processing

For whole-genome sequencing, large-insert SMRTBell libraries (> 20 kb), selected using a BluePippin System (Sage Science, Inc., Beverly, MA, USA), were prepared according to standard manufacture protocols. Libraries were sequenced using P6-C4 chemistry on either the RS II or Sequel sequencing instruments (Pacific BioSciences, Menlo Park, CA, USA; Additional file 13: Table S6). Sequencing was performed for *A. atlantica* at the DNA Sequencing Center (DNASC) at BYU (Provo, UT, USA) and at RTL Genomics (Lubbock, TX), while the sequencing for *A. eriantha* was performed at the BYU DNASC. For the diversity panel and for whole-genome polishing, extracted DNA was sent to the Beijing Genomic Institute (BGI; Hong Kong, China) for 2 × 150 bp paired end (PE) sequencing from standard 500-bp insert libraries. Trimmomatic v0.35 [105] was used to remove adapter sequences and leading and trailing bases with a quality score below 20 or average per-base quality of 20 over a four-nucleotide sliding window. After trimming, any reads shorter than 75 nucleotides in length were removed.

### RNA-Seq and transcriptome assembly

For *A. atlantica*, RNA-Seq data consisted 2 × 100 bp PE Illumina reads derived from 11 different plant tissue types including, stem, mature leaf, stressed mature leaf, seed (2 days old), hypocotyl (4–5 days old), root (4–5 days old), vegetative meristem, green grain, yellow grain, young flower (meiotic), and green anthers (Additional file 14: Table S7). For *A. eriantha*, 2 × 150 PE RNA-Seq data was generated by BGI from six tissue sources, including young leaf, mature leaf, crown, roots, immature panicle, and whole seedling, harvested from plants grown hydroponically in 1× Maxigro™ (GH Inc., Sebastopol, CA, USA) in growth chambers maintained at 21 °C under broad-spectrum halogen lamps, with a 12-h photoperiod at BYU (Additional file 14: Table S7). The resulting reads were trimmed using Trimmomatic [105], then aligned to either the *A. atlantica* or *A. eriantha* reference using HiSat2 v2.0.4 [106] with default parameters and max intron length set to 50,000 bp. Following alignment, the resulting SAM file was sorted and indexed using SAMtools v1.6 [107] and assembled into putative transcripts using StringTie v1.3.4 [108]. The quality of the assembled transcriptome was assessed relative to completeness using BLAST comparisons to the reference *Brachypodium distachyon* L. (ftp://ftp.ensemblgenomes.org/pub/plants/release-37/fasta/brachypodium_distachyon/pep/).

### Genome size, assembly, polishing, and scaffolding

Genome size was estimated using Jellyfish [109] and GenomeScope v1.0 [31] at k-mer length = 21 for each species. Initial assemblies were done using Canu v1.7 [33] with default parameters (corMhapSensitivity = normal and corOutCoverage = 40). The resulting assemblies were polished using Arrow from the GenomicConsensus package in the Pacific BioSciences SMRT portal v5.1.0 and PILON v0.22 [110] using Illumina short reads. Chicago® and Hi-C proximity-guided assemblies were performed by Dovetail Genomics LLC (Santa Cruz, CA, USA) to produce chromosome-scale assemblies. Fresh leaf tissue from a single dark-treated (72 h), 3-week-old plant, derived directly from selfing of the original *A. atlantica* and *A. eriantha* plants, was sent to Dovetail Genomics for Chicago® and Hi-C library preparation as described by Putnam et al. [35] and Lieberman-Aiden et al. [111], respectively, using the *Dpn*II restriction endonuclease. The libraries were sequenced using a standard Illumina library prep followed by sequencing on an Illumina HiSeq X in rapid run mode. The HiRiSE™ scaffolder and the Chicago® and Hi-C library-based read pairs were used to a produce a likelihood model for genomic distance between read pairs, which was used to break putative miss-joins and to identify and make prospective joins in the de novo Canu assemblies.

### Repeat analysis, genome completeness, and annotation

RepeatModeler v1.0.11 [112] and RepeatMasker v4.0.7 [113] were used to quantify and classify repetitive elements in the final assemblies, relative to RepBase libraries v20181026; www.girinst.org). Benchmarking Universal Single-Copy Orthologs (BUSCO) v3.0.2 [59] was employed to assess the completeness of the assembly using the Embryophyta odb9 dataset and the “—long” argument, which applies Augustus [114] optimization for self-training.

MAKER2 v2.31.10 [56, 57] was used to annotate the final genomes. Expressed sequence tag evidence for annotation included the de novo transcriptomes for each species as well as the cDNA models from *Brachypodium distachyon* L. (v 1.0; Ensembl genomes). Protein evidence included the uniprot-sprot database (downloaded September 25, 2018) as well as the peptide models from *B. distachyon* (v 1.0; Ensembl genomes). Repeats were masked based on species-specific files produced by RepeatModeler. For ab initio gene prediction, *A. atlantica* and *A. eriantha* species-specific AUGUSTUS gene prediction models were provided as well as rice (*Oryza sativa*)-based SNAP models.

### Variant identification and tree creation

Single nucleotide polymorphisms (SNPs) for the diversity panel were identified from the Illumina reads by mapping the A-subgenome and C-subgenome diploid accessions against the *A. atlantica* and *A. eriantha* reference genome assemblies, respectively, using BWA-mem v0.7.17 [115]. Output SAM files were converted to BAM files and sorted using SAMtools v1.6 [107], and indexed using Sambamba v0.6.8 [116]. InterSnp, an analysis tool from the BamBam v1.4 package [72], was used to call SNPs with the arguments -m 2 and -f 0.35. Bash scripting was used to removed SNPs with less than 100% genotype calls across all accessions (i.e., no missing data) or given the cleistogamous nature of the species where 5% or more of the accessions were called as heterozygotes. SNPs on unscaffolded contigs were also removed prior to phylogenetic analysis. SNPhylo v20160204 [73], which uses MUSCLE [117] for sequence alignments and linkage disequilibrium to down sample the SNP dataset, was used to build Phylogenies with the bootstrapping parameter set to 1000. The resulting tree was visualized using FigTree v1.4.3 (http://tree.bio.ed.ac.uk/software/figtree).

### Genome comparison

Genomic comparisons, including calculations of synonymous substitutions per synonymous sites (Ks) and homology searches for syntenic gene-sets with *Hordeum vulgare* L. (CoGe genome id52970 [118]), *Oryza sativa* L. (CoGe genome id34910 [119]), *Zea mays* L. (CoGe genome id33766 [120]), and *B. distachyon* (CoGe genome id52735 Vogel [121]) were accomplished using the DAGchainer output file from the CoGe (https://genomevolution.org/coge/) SynMap tool.

## Supplementary information

**Additional file 1:** **Table S1.** Summary of the repeat element content in the amaranth genome assembly as identified by RepeatMasker relative to the RepBase-derived RepeatMasker libraries.

**Additional file 2.** Telomeric satellite and centromeric repeat sequences.

**Additional file 3: Table S2.** List of Avena accessions included in the resequencing panel. Cc 7277 (*A. atlantica*) and CN 19328 (*A. eriantha*) were the reference genomes. The species and genome formula of each accession is presented. The raw read files can be found in BioProject PRJNA556219.

**Additional file 4:** **Table S3.** SNPs per chromosome use for maximum likelihood phylogeny produced using SNPhylo [73].

**Additional file 5: Figure S1.** Rate of synonymous substitutions per synonymous sites (Ks) within duplicated gene pairs from coding sequences predicted from *A. atlantica* comparisons with *A. eriantha*, *H. vulgare*, *B. distachyon*, *O. sativa*, and *Z. mays*.

**Additional file 6: Figure S2.** Orthologous genes were identified between *A. atlantica* and *A. eriantha* genomes to detect orthologous chromosome relationships. Genome synteny was (A) visualized by dotplot analysis, with boxes drawn around syntenic regions, (B) quantified, where the chromosome pairs with the highest amount of syntenic block connections, expressed as a percentage of the total syntenic bases, are colored red and transition to white as the number of connections decreases and (C) correlation between syntenic block sizes between *A. atlantica* and *A. eriantha*.

**Additional file 7: Figure S3.** Orthologous genes were identified between *A. atlantica* and *H. vulgare* genomes to detect orthologous chromosome relationships. Genome synteny was (A) visualized by dotplot analysis, with boxes drawn around syntenic regions, (B) quantified, where the chromosome pairs with the highest amount of syntenic block connections, expressed as a percentage of the total syntenic bases, are colored red and transition to white as the number of connections decreases and (C) correlation between syntenic block sizes between *A. atlantica* and *H. vulgare* (Hv_IBSC_PGSB_v2; Ensembl Release 36).

**Additional file 8: Figure S4.** Orthologous genes were identified between *A. eriantha* and *H. vulgare* genomes to detect orthologous chromosome relationships. Genome synteny was (A) visualized by dotplot analysis, with boxes drawn around syntenic regions, (B) quantified, where the chromosome pairs with the highest amount of syntenic block connections, expressed as a percentage of the total syntenic bases, are colored red and transition to white as the number of connections decreases and (C) correlation between syntenic block sizes between *A. eriantha* and *H. vulgare* (Hv_IBSC_PGSB_v2; Ensembl Release 36).

**Additional file 9: Table S4.** Summary of resistance gene analog identification results using the RGAugury pipeline [92] for *A. atlantica* and *A. eriantha*.

**Additional file 10: Figure S5.** Distribution of the resistance gene analogs (RGAs) encoding genes on the (A) *A. atlantica* and (B) *A. eriantha* genome. The RGAugury pipeline classifies RGA candidates into four major families based on the presence of RGA domains and motifs, specifically, nucleotide binding sites (NBS, blue), transmembrane coiled-coil (TMCC, black), and membrane associated receptor-like proteins kinases (RLK, yellow) and receptor-like proteins (RLP, green). The predicted location of the Pc91 crown rust QTLs (Klos et al. [30]) in the *A. atlantica* genome is shown on chromosome AA2.

**Additional file 11: Table S5.** Candidate resistance gene analogs associated with crown rust resistance on Mrg18 linkage group [28]. Mrg18 was previously shown to be involved in an intergenomic translocation involving 7C and 17A, corresponding to *A. eriantha* chromosome AE7 and *A. atlantica* chromosome AA2. Klos et al. [30] identified two QTLs associated *P. coronata* (crown rust) resistance on Mrg18, one of which determined to be Pc91. Candidate resistance gene analogs were identified using BLAST searches against the *A. atlantica* and *A. eriantha* genome assembly using makers sequences associated with the QTLs.

**Additional file 12: Figure S6.** Corresponding location of restriction fragment length polymorphism markers mapped on a segregating *A. strigosa* X *A. wiestii* population developed by Kremer et al. [102] on the *A. atlantica* chromosomes (shown on a gene density plot). Markers from each of their linkage groups (Asw A-I) are color-coded with approximate positions on *A. atlantica* scaffolds indicated with arrows.

**Additional file 13:** **Table S6.** PacBio and Illumina sequencing read statistics. The raw read files can be found in BioProjects PRJNA546592 and PRJNA546595.

**Additional file 14: Table S7.** Raw read statistics for RNASeq data for *A. atlantica* and *A. eriantha*. All reads were illumina pair-end reads from standard 500 bp insert libraries. The raw read files can be found in BioProjects PRJNA556794 and PRJNA546595.

**Additional file 15: Figure S7.** Unabbreviated A-genome diploids rooted to the *A. eriantha* reference (ER_CN 19238). Accession names are abbreviated as described in Additional file 3: Table S2.

**Additional file 16: Figure S8.** Unabbreviated C-genome diploids rooted to the *A. atlantica* (AT_Cc 7277) reference. Accession names are abbreviated as described in Additional file 3: Table S2.

## Data Availability

All data generated or analyzed during this study are included in this published article, its supplementary information files and publicly available repositories. The raw sequences used for *A. atlantica* genome assembly are deposited in the National Center for Biotechnology Information (NCBI) Sequence Read Archive database under the BioProject PRJNA546592 [122] with the following accession numbers: SRR9720684 (PacBio reads), SRR9841448–SRR9841455 (Hi-C reads), SRR9841587–SRR9841597 (Transcriptome). Similarly, the raw sequences for the *A. eriantha* genome assembly are found in BioProject PRJNA546595 [123] with the following accession numbers: SRR9720373 (PacBio reads), SRR9833273–SRR9833276 (Hi-C reads), SRR9722223 (Polishing short reads), SRR9722219–SRR9722222, SRR9722225, SRR9722226 (Transcriptome). The raw reads for the resequencing panel of the diploid species (Additional file 3: Table S2) are found in BioProject PRJNA556219 [124] with the following NCBI accession numbers: SRR9933122–SRR9933198 (resequencing panel). Genome browsing and bulk data downloads, including annotations and BLAST analysis of the final proximity-guided assemblies are available at CoGe (https://genomevolution.org/coge/) with genome IDs: id53337 (*A. atlantica* [125]) and id53381 (*A. eriantha* [126]).

## Abbreviations

BLAST: Basic Local Alignment Search Tool
GWAS: Genome-wide association studies
LG: Linkage group
Ma: Million years ago
QTL: Quantitative trail loci
RGA: Resistance gene analog
SNPs: Single nucleotide polymorphisms

## References

[1] Ahmad M, Zaffar G, Mir SD, Razvi SM, Dar ZA, Iqbal S (2014). Genetic analysis for fodder yield and other important traits in oats (Avena sativa L.). Indian J Genet Pl Br.

[2] Oliver RE, Tinker NA, Lazo GR, Chao S, Jellen EN, Carson ML, Rines HW, Obert DE, Lutz JD, Shackelford I (2013). SNP discovery and chromosome anchoring provide the first physically-anchored hexaploid oat map and reveal synteny with model species. PLoS One.

[3] Andon MB, Anderson JW (2008). State of the art reviews: the oatmeal-cholesterol connection: 10 years later. Am J Lifestyle Med.

[4] Jenkins AL, Jenkins DJA, Zdravkovic U, Wursch P, Vuksan V (2002). Depression of the glycemic index by high levels of beta-glucan fiber in two functional foods tested in type 2 diabetes. Eur J Clin Nutr.

[5] Yarnell E, Abascal K (2001). Botanical remedies for nicotine addiction. Altern Complement Ther.

[6] Yarnell EaA K (2001). Botanical treatments for depression. Altern Complement Ther.

[7] Fardet A (2010). New hypotheses for the health-protective mechanisms of whole-grain cereals: what is beyond fibre?. Nutr Res Rev.

[8] Peterson DM (2001). Oat antioxidants. J Cereal Sci.

[9] Daou C, Zhang H (2012). Oat Beta-Glucan: its role in health promotion and prevention of diseases. Compr Rev Food Sci F.

[10] Grimalt R, Mengeaud V, Cambazard F, Study Investigators G (2007). The steroid-sparing effect of an emollient therapy in infants with atopic dermatitis: a randomized controlled study. Dermatology.

[11] Potter RC, Castro JM, L.C. M: Oat oil compositions with useful cosmetic and dermatological properties in*.* Edited by States U, vol. US5620692A. United States: GTC OATS Inc 1997.

[12] Singh R, De S, Belkheir A (2013). Avena sativa (oat), a potential Neutraceutical and therapeutic agent: an overview. Crit Rev Food Sci.

[13] Sur R, Nigam A, Grote D, Liebel F, Southall MD (2008). Avenanthramides, polyphenols from oats, exhibit anti-inflammatory and anti-itch activity. Arch Dermatol Res.

[14] Soreng RJ, Peterson PM, Romaschenko K, Davidse G, Teisher JK, Clark LG, Barbera P, Gillespie LJ, Zuloaga FO (2017). A worldwide phylogenetic classification of the Poaceae (Gramineae) II: an update and a comparison of two 2015 classifications. J Syst Evol.

[15] Zhou X, Jellen EN, Murphy JP (1999). Progenitor germplasm of domesticated hexaploid oat. Crop Sci.

[16] Yan H, Bekele WA, Wight CP, Peng Y, Langdon T, Latta RG, Fu YB, Diederichsen A, Howarth CJ, Jellen EN (2016). High-density marker profiling confirms ancestral genomes of Avena species and identifies D-genome chromosomes of hexaploid oat. Theor Appl Genet.

[17] Loskutov IG, Rines HW, Kole C (2011). Avena. Wild Crop Relatives: Genomic and Breeding Resources.

[18] Aung T, Chong J, Leggett M. The transfer of crown rust resistance Pc94 from a wild diploid to cultivated hexaploid oat. In: Kema GHJ, Niks RE, Daamen RA (eds) Proc. 9th Int. Eur. Mediterr. Cereal Rusts and Powdery Mildews Conf. Lunteren Netherlands. Wageningen, European and Mediterranean Cereal Rust Foundation. 1996. pp. 167–71.

[19] Dyck PL, Zillinsky FJ (1963). Inheritance of Crown Rust Resistance Transferred from Diploid to Hexaploid Oats. Can J Genet Cytol.

[20] Welch RW, Brown JCW, Leggett JM (2000). Interspecific and intraspecific variation in grain and great characteristics of wild oat (Avena) species: Very high great (1 -> 3),(1 -> 4)-beta-D-glucan in an Avena atlantica genotype. J Cereal Sci.

[21] Jellen EN, Beard J (2000). Geographical distribution of a chromosome 7C and 17 intergenomic translocation in cultivated oat. Crop Sci.

[22] Fominaya A, Vega C, Ferrer E (1988). Giemsa C-banded karyotypes of Avena species. Genome.

[23] Coon MA (2012). Characterization and variable expression of the CslF6 homologs in oat (*Avena* sp.).

[24] Jellen EN, Gill BS, Cox TS (1994). Genomic in-situ hybridization differentiates between a/D-genome and C-genome chromatin and detects Intergenomic translocations in Polyploid oat species (genus Avena). Genome.

[25] Oliver RE, Jellen EN, Ladizinsky G, Korol AB, Kilian A, Beard JL, Dumlupinar Z, Wisniewski-Morehead NH, Svedin E, Coon M (2011). New diversity arrays technology (DArT) markers for tetraploid oat (Avena magna Murphy et Terrell) provide the first complete oat linkage map and markers linked to domestication genes from hexaploid a. sativa L. Theor Appl Genet.

[26] Bennett MD, Smith JB (1976). Nuclear dna amounts in angiosperms. Philos Trans R Soc Lond Ser B Biol Sci.

[27] Sanz MJ, Jellen EN, Loarce Y, Irigoyen ML, Ferrer E, Fominaya A (2010). A new chromosome nomenclature system for oat (Avena sativa L. and A. byzantina C. Koch) based on FISH analysis of monosomic lines. Theor Appl Genet.

[28] Chaffin AS, Huang YF, Smith S, Bekele WA, Babiker E, Gnanesh BN, Foresman BJ, Blanchard SG, Jay JJ, Reid RW et al. A Consensus Map in Cultivated Hexaploid Oat Reveals Conserved Grass Synteny with Substantial Subgenome Rearrangement. Plant Genome. 2016;9(2):1–21. 10.3835/plantgenome2015.10.0102 27898818

[29] Bekele WA, Wight CP, Chao SM, Howarth CJ, Tinker NA (2018). Haplotype-based genotyping-by-sequencing in oat genome research. Plant Biotechnol J.

[30] Klos KE, Yimer BA, Babiker EM, Beattie AD, Bonman JM, Carson ML, Chong J, Harrison SA, Ibrahim AMH, Kolb FL *et al*. Genome-Wide Association Mapping of Crown Rust Resistance in Oat Elite Germplasm. Plant Genome. 2017;10(2):1–13. 10.3835/plantgenome2016.10.0107 28724060

[31] Vurture GW, Sedlazeck FJ, Nattestad M, Underwood CJ, Fang H, Gurtowski J, Schatz MC (2017). GenomeScope: fast reference-free genome profiling from short reads. Bioinformatics.

[32] Yan H, Martin SL, Bekele WA, Latta RG, Diederichsen A, Peng Y, Tinker NA (2016). Genome size variation in the genus Avena. Genome.

[33] Koren S, Walenz BP, Berlin K, Miller JR, Bergman NH, Phillippy AM (2017). Canu: scalable and accurate long-read assembly via adaptive k-mer weighting and repeat separation. Genome Res.

[34] Singh R, Ming R, Yu QY (2016). Comparative analysis of GC content variations in plant genomes. Trop Plant Biol.

[35] Putnam NH, O'Connell BL, Stites JC, Rice BJ, Blanchette M, Calef R, Troll CJ, Fields A, Hartley PD, Sugnet CW (2016). Chromosome-scale shotgun assembly using an in vitro method for long-range linkage. Genome Res.

[36] Latta RG, Bekele WA, Wight CP, Tinker NA: Comparative linkage mapping of diploid, tetraploid, and hexaploid Avena species suggests extensive chromosome rearrangement in ancestral diploids. Sci Rep 2019, In Press. 10.1038/s41598-019-48639-7 PMC6707241 31444367

[37] Tinker NA, Bekele WA, Hattori J (2016). Haplotag: Software for Haplotype-Based Genotyping-by-Sequencing Analysis. G3-Genes Genom Genet.

[38] Haupt W, Fischer TC, Winderl S, Fransz P, Torres-Ruiz RA (2001). The centromere1 (CEN1) region of Arabidopsis thaliana: architecture and functional impact of chromatin. Plant J.

[39] Talbert PB, Henikoff S (2010). Centromeres convert but don't cross. PLoS Biol.

[40] Du J, Tian Z, Hans CS, Laten HM, Cannon SB, Jackson SA, Shoemaker RC, Ma J (2010). Evolutionary conservation, diversity and specificity of LTR-retrotransposons in flowering plants: insights from genome-wide analysis and multi-specific comparison. Plant J.

[41] Galindo-Gonzalez L, Mhiri C, Deyholos MK, Grandbastien MA (2017). LTR-retrotransposons in plants: engines of evolution. Gene.

[42] Tenaillon MI, Hollister JD, Gaut BS (2010). A triptych of the evolution of plant transposable elements. Trends Plant Sci.

[43] Ibarra-Laclette E, Lyons E, Hernandez-Guzman G, Perez-Torres CA, Carretero-Paulet L, Chang TH, Lan T, Welch AJ, Juarez MJ, Simpson J (2013). Architecture and evolution of a minute plant genome. Nature.

[44] Schnable PS, Ware D, Fulton RS, Stein JC, Wei F, Pasternak S, Liang C, Zhang J, Fulton L, Graves TA (2009). The B73 maize genome: complexity, diversity, and dynamics. Science.

[45] Tian ZX, Rizzon C, Du JC, Zhu LC, Bennetzen JL, Jackson SA, Gaut BS, Ma JX (2009). Do genetic recombination and gene density shape the pattern of DNA elimination in rice long terminal repeat retrotransposons?. Genome Res.

[46] Paterson AH, Bowers JE, Bruggmann R, Dubchak I, Grimwood J, Gundlach H, Haberer G, Hellsten U, Mitros T, Poliakov A (2009). The Sorghum bicolor genome and the diversification of grasses. Nature.

[47] Baucom RS, Estill JC, Chaparro C, Upshaw N, Jogi A, Deragon JM, Westerman RP, Sanmiguel PJ, Bennetzen JL (2009). Exceptional diversity, non-random distribution, and rapid evolution of retroelements in the B73 maize genome. PLoS Genet.

[48] Langdon T, Jenkins G, Hasterok R, Jones RN, King LP (2003). A high-copy-number CACTA family transposon in temperate grasses and cereals. Genetics.

[49] Wicker T, Guyot R, Yahiaoui N, Keller B (2003). CACTA transposons in Triticeae. A diverse family of high-copy repetitive elements. Plant Physiol.

[50] Bilinski P, Han Y, Hufford MB, Lorant A, Zhang P, Estep MC, Jiang J, Ross-Ibarra J (2017). Genomic abundance is not predictive of tandem repeat localization in grass genomes. PLoS One.

[51] Feschotte C, Swamy L, Wessler SR (2003). Genome-wide analysis of mariner-like transposable elements in rice reveals complex relationships with stowaway miniature inverted repeat transposable elements (MITEs). Genetics.

[52] Minaya M, Pimentel M, Mason-Gamer R, Catalan P (2013). Distribution and evolutionary dynamics of stowaway miniature inverted repeat transposable elements (MITEs) in grasses. Mol Phylogenet Evol.

[53] Solano R, Hueros G, Fominaya A, Ferrer E (1992). Organization of repeated sequences in species of the genus Avena. Theor Appl Genet.

[54] Katsiotis A, Loukas M, Heslop-Harrison JS (2000). Repetitive DNA, genome and species relationships in Avena and Arrhenatherum (Poaceae). Ann Bot-London.

[55] Michael TP (2014). Plant genome size variation: bloating and purging DNA. Brief Funct Genomics.

[56] Cantarel BL, Korf I, Robb SM, Parra G, Ross E, Moore B, Holt C, Sanchez Alvarado A, Yandell M (2008). MAKER: an easy-to-use annotation pipeline designed for emerging model organism genomes. Genome Res.

[57] Holt C, Yandell M (2011). MAKER2: an annotation pipeline and genome-database management tool for second-generation genome projects. BMC Bioinformatics.

[58] Duret L, Galtier N (2009). Biased gene conversion and the evolution of mammalian genomic landscapes. Annu Rev Genomics Hum Genet.

[59] Simao FA, Waterhouse RM, Ioannidis P, Kriventseva EV, Zdobnov EM (2015). BUSCO: assessing genome assembly and annotation completeness with single-copy orthologs. Bioinformatics.

[60] Mizuno H, Kawahara Y, Wu JZ, Katayose Y, Kanamori H, Ikawa H, Itoh T, Sasaki T, Matsumoto T. Asymmetric distribution of gene expression in the centromeric region of rice chromosome 5. Front Plant Sci. 2011;2(16)1–12. 10.3389/fpls.2011.00016 PMC3355683 22639581

[61] Philippe R, Paux E, Bertin I, Sourdille P, Choulet F, Laugier C, Simkova H, Safar J, Bellec A, Vautrin S *et al*: A high density physical map of chromosome 1BL supports evolutionary studies, map-based cloning and sequencing in wheat. Genome Biol. 2013;14(6):1–22. 10.1186/gb-2013-14-6-r64 PMC4054855 23800011

[62] Henikoff S, Ahmad K, Malik HS (2001). The centromere paradox: stable inheritance with rapidly evolving DNA. Science.

[63] Melters DP, Bradnam KR, Young HA, Telis N, May MR, Ruby JG, Sebra R, Peluso P, Eid J, Rank D *et al*: Comparative analysis of tandem repeats from hundreds of species reveals unique insights into centromere evolution. Genome Biol. 2013;14(1):1–20. 10.1186/gb-2013-14-1-r10 PMC4053949 23363705

[64] Willing EM, Rawat V, Mandakova T, Maumus F, James GV, Nordstrom KJV, Becker C, Warthmann N, Chica C, Szarzynska B (2015). Genome expansion of Arabis alpina linked with retrotransposition and reduced symmetric DNA methylation. Nat Plants.

[65] Gan X, Hay A, Kwantes M, Haberer G, Hallab A, Dello Ioio R, Hofhuis H, Pieper B, Cartolano M, Neumann U *et al*: The *Cardamine hirsuta* genome offers insight into the evolution of morphological diversity. Nat Plants. 2016;2(11):1–6. 10.1038/nplants.2016.167 PMC8826541 27797353

[66] Baum BR, Fedak G (1985). Avena-Atlantica, a new diploid species of the oat genus from Morocco. Can J Bot.

[67] Rajhathy T, Dyck PL (1963). Chromosomal Differentiation and Speciation in Diploid Avena .2. Karyotype of *A. pilosa*. Can J Genet Cytol.

[68] Schubert I, Schrieverschwemmer G, Werner T, Adler ID (1992). Telomeric signals in Robertsonian fusion and fission chromosomes - implications for the origin of Pseudoaneuploidy. Cytogenet Cell Genet.

[69] Islam-Faridi MN, Nelson CD, Kubisiak TL (2007). Reference karyotype and cytomolecular map for loblolly pine (Pinus taeda L.). Genome.

[70] Raskina O, Barber JC, Nevo E, Belyayev A (2008). Repetitive DNA and chromosomal rearrangements: speciation-related events in plant genomes. Cytogenet Genome Res.

[71] Sousa A, Cusimano N, Renner SS (2014). Combining FISH and model-based predictions to understand chromosome evolution in Typhonium (Araceae). Ann Bot.

[72] Page JT, Liechty ZS, Huynh MD, Udall JA (2014). BamBam: genome sequence analysis tools for biologists. BMC Res Notes.

[73] Lee TH, Guo H, Wang X, Kim C, Paterson AH (2014). SNPhylo: a pipeline to construct a phylogenetic tree from huge SNP data. BMC Genomics.

[74] Rajhathy T, Morrison JW (1959). Chromosome morphology in the genus *Avena*. Can J Bot.

[75] Leggett JM (1987). Interspecific hybrids involving the recently described diploid taxon Avena-Atlantica. Genome.

[76] Bremer K (1992). Ancestral areas - a Cladistic reinterpretation of the Center of Origin Concept. Syst Biol.

[77] Bouchenak-Khelladi Y, Verboom GA, Savolainen V, Hodkinson TR (2010). Biogeography of the grasses (Poaceae): a phylogenetic approach to reveal evolutionary history in geographical space and geological time. Bot J Linn Soc.

[78] Schubert M, Marcussen T, Meseguer AS, Fjellheim S (2019). The grass subfamily Pooideae: cretaceous-Palaeocene origin and climate-driven Cenozoic diversification. Glob Ecol Biogeogr.

[79] Christin PA, Spriggs E, Osborne CP, Stromberg CAE, Salamin N, Edwards EJ (2014). Molecular dating, evolutionary rates, and the age of the grasses. Syst Biol.

[80] Yang Z (2007). PAML 4: phylogenetic analysis by maximum likelihood. Mol Biol Evol.

[81] Koch MA, Haubold B, Mitchell-Olds T (2000). Comparative evolutionary analysis of chalcone synthase and alcohol dehydrogenase loci in Arabidopsis, Arabis, and related genera (Brassicaceae). Mol Biol Evol.

[82] Lynch M, Conery JS (2000). The evolutionary fate and consequences of duplicate genes. Science.

[83] Mascher M, Gundlach H, Himmelbach A, Beier S, Twardziok SO, Wicker T, Radchuk V, Dockter C, Hedley PE, Russell J (2017). A chromosome conformation capture ordered sequence of the barley genome. Nature.

[84] Jellen EN, Gill BS, Rines HW, Fox SL, Wilson WA, McMullen MS. Translocations in current and ancestral spring and winter oat accessions. In: 1996 Agronomy abstracts, vol. 1996. Madison: Agronomy Society of America. p. 78.

[85] Jarvis DE, Ho YS, Lightfoot DJ, Schmockel SM, Li B, Borm TJA, Ohyanagi H, Mineta K, Michell CT, Saber N (2017). The genome of *Chenopodium quinoa*. Nature.

[86] Du XM, Huang G, He SP, Yang ZE, Sun GF, Ma XF, Li N, Zhang XY, Sun JL, Liu M (2018). Resequencing of 243 diploid cotton accessions based on an updated A genome identifies the genetic basis of key agronomic traits. Nat Genet.

[87] Bertioli DJ, Cannon SB, Froenicke L, Huang GD, Farmer AD, Cannon EKS, Liu X, Gao DY, Clevenger J, Dash S (2016). The genome sequences of Arachis duranensis and Arachis ipaensis, the diploid ancestors of cultivated peanut. Nat Genet.

[88] Mathan J, Bhattacharya J, Ranjan A (2016). Enhancing crop yield by optimizing plant developmental features. Development.

[89] Yan L, Fu D, Li C, Blechl A, Tranquilli G, Bonafede M, Sanchez A, Valarik M, Yasuda S, Dubcovsky J (2006). The wheat and barley vernalization gene VRN3 is an orthologue of FT. P Natl Acad Sci USA.

[90] Carson ML (2011). Virulence in oat crown rust (Puccinia coronata f. sp avenae) in the United States from 2006 through 2009. Plant Dis.

[91] Simmons MD (1985). The Cereal Rusts Vol II: Diseases, distribution, epidemiology and control.

[92] Li PC, Quan XD, Jia GF, Xiao J, Cloutier S, You FM. RGAugury: a pipeline for genome-wide prediction of resistance gene analogs (RGAs) in plants. BMC Genomics. 2016;17(852):1–10. 10.1186/s12864-016-3197-x PMC5093994 27806688

[93] Akita M, Valkonen JPT (2002). A novel gene family in moss (Physcomitrella patens) shows sequence homology and a phylogenetic relationship with the TIR-NBS class of plant disease resistance genes. J Mol Evol.

[94] Bai J, Pennill LA, Ning J, Lee SW, Ramalingam J, Webb CA, Zhao B, Sun Q, Nelson JC, Leach JE (2002). Diversity in nucleotide binding site-leucine-rich repeat genes in cereals. Genome Res.

[95] McDowell JM, Dhandaydham M, Long TA, Aarts MGM, Goff S, Holub EB, Dangl JL (1998). Intragenic recombination and diversifying selection contribute to the evolution of downy mildew resistance at the RPP8 locus of arabidopsis. Plant Cell.

[96] Grant MR, Godiard L, Straube E, Ashfield T, Lewald J, Sattler A, Innes RW, Dangl JL (1995). Structure of the Arabidopsis Rpm1 gene enabling dual-specificity disease resistance. Science.

[97] McCartney CA, Stonehouse RG, Rossnagel BG, Eckstein PE, Scoles GJ, Zatorski T, Beattie AD, Chong J (2011). Mapping of the oat crown rust resistance gene Pc91. Theor Appl Genet.

[98] Gnanesh BN, Fetch JM, Menzies JG, Beattie AD, Eckstein PE, McCartney CA (2013). Chromosome location and allele-specific PCR markers for marker-assisted selection of the oat crown rust resistance gene Pc91. Mol Breeding.

[99] Garcia-Castellanos D, Estrada F, Jimenez-Munt I, Gorini C, Fernandez M, Verges J, De Vicente R (2009). Catastrophic flood of the Mediterranean after the Messinian salinity crisis. Nature.

[100] Blanc PL (2002). The opening of the Plio-quaternary Gibraltar Strait: assessing the size of a cataclysm. Geodin Acta.

[101] Kropelin S, Verschuren D, Lezine AM, Eggermont H, Cocquyt C, Francus P, Cazet JP, Fagot M, Rumes B, Russell JM (2008). Climate-driven ecosystem succession in the Sahara: the past 6000 years. Science.

[102] Kremer CA, Lee M, Holland JB (2001). A restriction fragment length polymorphism based linkage map of a diploid Avena recombinant inbred line population. Genome.

[103] Sambrook J, Fritsch EF, Maniatis T (1989). Molecular cloning: A laboratory manual.

[104] Todd JJ, Vodkin LO (1996). Duplications that suppress and deletions that restore expression from a Chalcone synthase multigene family. Plant Cell.

[105] Bolger AM, Lohse M, Usadel B (2014). Trimmomatic: a flexible trimmer for Illumina sequence data. Bioinformatics.

[106] Kim D, Langmead B, Salzberg SL (2015). HISAT: a fast spliced aligner with low memory requirements. Nat Methods.

[107] Li H, Handsaker B, Wysoker A, Fennell T, Ruan J, Homer N, Marth G, Abecasis G, Durbin R, Genome project data processing S (2009). The sequence alignment/map format and SAMtools. Bioinformatics.

[108] Pertea M, Kim D, Pertea GM, Leek JT, Salzberg SL (2016). Transcript-level expression analysis of RNA-seq experiments with HISAT, StringTie and Ballgown. Nat Protoc.

[109] Marcais G, Kingsford C (2011). A fast, lock-free approach for efficient parallel counting of occurrences of k-mers. Bioinformatics.

[110] Walker BJ, Abeel T, Shea T, Priest M, Abouelliel A, Sakthikumar S, Cuomo CA, Zeng Q, Wortman J, Young SK (2014). Pilon: an integrated tool for comprehensive microbial variant detection and genome assembly improvement. PLoS One.

[111] Lieberman-Aiden E, van Berkum NL, Williams L, Imakaev M, Ragoczy T, Telling A, Amit I, Lajoie BR, Sabo PJ, Dorschner MO (2009). Comprehensive mapping of long-range interactions reveals folding principles of the human genome. Science.

[112] Smit A, Hubley, R: RepeatModeler Open-1.0. 2008-2015, <http://www.repeatmasker.org>. Accessed 22 Apr 2019.

[113] Smit AFA, Hubley R, Green P: RepeatMasker Open-4.0. 2013-2015 <http://www.repeatmasker.org>. Accessed 22 Apr 2019.

[114] Stanke M, Tzvetkova A, Morgenstern B (2006). AUGUSTUS at EGASP: using EST, protein and genomic alignments for improved gene prediction in the human genome. Genome Biol.

[115] Li H: Aligning sequence reads, clone sequences and assembly contigs with BWA-MEM. *arXiv* 2013, preprint arXiv:1303.3997.

[116] Tarasov A, Vilella AJ, Cuppen E, Nijman IJ, Prins P (2015). Sambamba: fast processing of NGS alignment formats. Bioinformatics.

[117] Edgar RC (2004). MUSCLE: multiple sequence alignment with high accuracy and high throughput. Nucleic Acids Res.

[118] Mayer KF, Waugh R, Brown JW, Schulman A, Langridge P. A physical, genetic and functional sequence assembly of the barley genome. Nature. 2012;491. 10.1038/nature11543 23075845

[119] Du HL, Yu Y, Ma YF, Gao Q, Cao YH, Chen Z, Ma B, Qi M, Li Y, Zhao XF, et al. Sequencing and de novo assembly of a near complete indica rice genome. Nat Commun. 2017;8. 10.1038/ncomms15324 PMC5418594 28469237

[120] Jiao YP, Peluso P, Shi JH, Liang T, Stitzer MC, Wang B, Campbell MS, Stein JC, Wei XH, Chin CS (2017). Improved maize reference genome with single-molecule technologies. Nature.

[121] Vogel JP, Garvin DF, Mockler TC, Schmutz J, Rokhsar D, Bevan MW, Barry K, Lucas S, Harmon-Smith M, Lail K (2010). Genome sequencing and analysis of the model grass Brachypodium distachyon. Nature.

[122] Maughan PJ, Lee R, Walstead RN, Vickerstaff RJ, Fogarty MC, Brouwer CR, Reid RR, Jay JJ, Bekele WA, Jackson EW *et al*: Raw sequences used for *A. atlantica* genome assembly are deposited in the Sequence Read Archive database under the BioProject PRJNA546592. National Center for Biotechnology Information; 2019: https://www.ncbi.nlm.nih.gov/bioproject/PRJNA546592. Accessed 5 Jun 2019.

[123] Maughan PJ, Lee R, Walstead RN, Vickerstaff RJ, Fogarty MC, Brouwer CR, Reid RR, Jay JJ, Bekele WA, Jackson EW *et al*: Raw sequences used for *A. eriantha* genome assembly are deposited in the Sequence Read Archive database under the BioProject PRJNA546595. National Center for Biotechnology Information; 2019: https://www.ncbi.nlm.nih.gov/bioproject/PRJNA546595. Accessed 5 Jun 2019.

[124] Maughan PJ, Lee R, Walstead RN, Vickerstaff RJ, Fogarty MC, Brouwer CR, Reid RR, Jay JJ, Bekele WA, Jackson EW *et al*: The raw reads for the resequencing panel of the Avena diploid species are found in BioProject PRJNA556219**.** National Center for Biotechnology Information; 2019: https://www.ncbi.nlm.nih.gov/bioproject/PRJNA556219. Accessed 23 Jul 2019.

[125] Maughan PJ, Lee R, Walstead RN, Vickerstaff RJ, Fogarty MC, Brouwer CR, Reid RR, Jay JJ, Bekele WA, Jackson EW *et al*: Avena atlantica genome and annotation**.** Comparative Genomics; 2019: https://genomevolution.org/coge/GenomeInfo.pl?gid=53337. Accessed 21 Dec 2018.

[126] Maughan PJ, Lee R, Walstead RN, Vickerstaff RJ, Fogarty MC, Brouwer CR, Reid RR, Jay JJ, Bekele WA, Jackson EW *et al*: Avena eriantha genome and annotation**.** Comparative Genomics; 2019: https://genomevolution.org/coge/GenomeInfo.pl?gid=53381. Accessed 2 Jan 2019.

