# Supplementary material for: Genomic insights from the first chromosome-scale assemblies of oat (Avena spp.) diploid species
Source: BMC Biol. 2019 Nov 22;17:92. doi: 10.1186/s12915-019-0712-y (PMC6874827; doi:10.1186/s12915-019-0712-y)
Supplement: Supplementary file 1 — Additional file 1: Table S1. Summary of the repeat element content in the amaranth genome assembly as identified by RepeatMasker relative to the RepBase-derived RepeatMasker libraries. [file 12915_2019_712_MOESM1_ESM.docx]

**Additional file 1: Table S1.** Summary of the repeat element content in the amaranth genome assembly as identified by RepeatMasker relative to the RepBase-derived RepeatMasker libraries.

| **Repeat Class^1^** | ***A. atlantica*** (3,673,044,503 bp) | | | ***A. eriantha*** (3,776,743,233 bp) | | |
| --- | --- | --- | --- | --- | --- | --- |
|  | Count | Bases masked | Masked | Count | Bases masked | Masked |
| **DNA** | 12343 | 2295245 | 0.06% | 41071 | 13892671 | 0.37% |
| CMC-EnSpm | 176565 | 183627714 | 5.00% | 235692 | 181347535 | 4.80% |
| MULE-MuDR | 16890 | 5211297 | 0.14% | 13913 | 9147023 | 0.24% |
| MuLE-MuDR | 6397 | 5618469 | 0.15% | 13476 | 18601079 | 0.49% |
| Maverick | 148 | 18931 | 0.00% | -- | -- | -- |
| PIF-Harbinger | 34425 | 10378659 | 0.28% | 53235 | 28107529 | 0.74% |
| TcMar-Stowaway | 82949 | 13125880 | 0.36% | 108832 | 20258806 | 0.54% |
| hAT-Ac | 2924 | 849064 | 0.02% | 6590 | 2464996 | 0.07% |
| hAT-Tag1 | 878 | 410769 | 0.01% | 5261 | 3946889 | 0.10% |
| hAT-Tip100 | 1561 | 651355 | 0.02% | 1875 | 932660 | 0.02% |
| **LINE** | -- | -- | -- | -- | -- | -- |
| CR1 | 922 | 101045 | 0.00% | -- | -- | -- |
| Jockey | 145 | 30396 | 0.00% | 5977 | 4017019 | 0.11% |
| L1 | 42540 | 33266727 | 0.91% | 44555 | 36045210 | 0.95% |
| R1 | 1345 | 438210 | 0.01% | -- | -- | -- |
| L2 | -- | -- | -- | 573 | 326088 | 0.01% |
| RTE-X | -- | -- | -- | 1738 | 877096 | 0.02% |
| **LTR** | 32110 | 49218294 | 1.34% | 14612 | 5824958 | 0.15% |
| Copia | 312901 | 641161159 | 17.46% | 254114 | 522841719 | 13.84% |
| Gypsy | 705163 | 1758990581 | **47.89%** | 715788 | 1829333860 | **48.44%** |
| Viper | -- | -- | -- | 469 | 281891 | 0.01% |
| **RC** | -- | -- | -- | -- | -- | -- |
| Pao | 519 | 285086 | 0.01% | -- | -- | -- |
| Helitron | -- | -- | -- | 1695 | 568721 | 0.02% |
| L1 | 10983 | 4727496 | 0.13% | 8142 | 1669307 | 0.04% |
| tRNA | 3080 | 530660 | 0.01% | 6237 | 4121298 | 0.11% |
| **Unknown** | 533080 | 322656906 | 8.78% | 693112 | 447222379 | 11.84% |
| **Total interspersed** | 1977868 | 3033593943 | 82.59% | 2226957 | 3131828734 | 82.92% |
| Low_complexity | 22741 | 1212274 | 0.03% | 21382 | 1166133 | 0.03% |
| Satellite | 5217 | 2364614 | 0.06% | 3404 | 943623 | 0.02% |
| Telomeric repeat | -- | -- | -- | 1815 | 14459837 | 0.38% |
| Simple repeat | 176100 | 10467715 | 0.28% | 162410 | 10363028 | 0.27% |
| Total | 2181926 | 3047638546 | 82.97% | 2415968 | 3158761355 | 83.64% |

^1^SINE, short interspersed nuclear elements; LINE, long interspersed nuclear elements; LTR, long terminal repeat; RC, Rolling circle

^2^The full sequence of the telomeric repeat is provided in additional file 2
