# Supplementary material for: Genomic insights from the first chromosome-scale assemblies of oat (Avena spp.) diploid species
Source: BMC Biol. 2019 Nov 22;17:92. doi: 10.1186/s12915-019-0712-y (PMC6874827; doi:10.1186/s12915-019-0712-y)
Supplement: Supplementary file 2 — Additional file 2. Telomeric satellite and centromeric repeat sequences. [file 12915_2019_712_MOESM2_ESM.docx]

**Additional file 2.** Telomeric satellite and centromeric repeat sequences.

*A. eriantha* telomeric satellite repeat

CTCAAACNTGTATCGGGTCTTATGGNCGATGNAAATCGCNTGGAACCCCAAAACTGTGGGCAATACCTCATGAAAACGGCCATAAAACGCGAAAACTACGAGTTTCGTGTCATAACATGTATCGGGTCTTACGGTCNTTGTAAATCNCCCTAGAACCCCAAAACCGTGAGCAATAGCTCATGAAAACGGCCATAAAACGCGAAAAAGACGAGTTTTTGGTCATATCTCTCAAACATGTATCGGGTCTTACGNTCGTTGTAAATCGCCNTGGAACCCCAAAATTGTGGGCAATAGCTCATGAAAACGGCCATAAAACGCTGAAACATGTATCGGGTCTTACGGTCGTTGTAAATNNCCCTAGAACCCCAAAACTGTGGGCAATAGCTCATGAAAACGGCCATAAAACGCGAAAACGACGAGTTTTTGGTCATATCTCTCAAACATGTATCGGGTCTTACGGTCGTTGTAAATCGCCCTGAAACCCCAAAACTGTGGGCAATAGCTCATGAAAACGGCCATAAAGCGCGAAAACGACGAGTTTTTGGTCATATCTCTCAAACNTGTATCGGGTCTTACGGTCNNTGNAAATCGCCTGGAACCCCAAAACTGTGGGCAATAGCTCATGAAAACGGCCATAAAACGCNAAAACTACGAGTTTTTGTCAT

*A. atlantica* telomeric satellite repeat

CTCAAACATGTATCGTGTCTTGCTGTCATTTTAAATCGCCCTGGAACACCAANANTATGGGCAATAACTCATGAAAACGGCCATAAAACGCGAAAACGACGAGTTCTTGGTCATGACTCTCAAACATGTAAATCGCCTTGGAACCCCAAAACTGTGGGCAATANCTCNTGAAAACGGCCNTAAAACACGAAAATGGAGAGTTTTTGGTCATGCCNTCAAACATGTATCGGGTCTTACGGTCATTTTAAATCGCCCTGGAACCCCAATATTATGGGCAATAACTCATGAAAACGGCCATAAAACGCGAAAACAACGAGTGCTTGGTCATAACTCTCAAACATGTAAATCGCCTTGGAACCCCAAAACTGTGGGCAATNGCTCATGAAAACGGCCATAAAACACGAAAATGGAGAGTTTTTGGTCATGCCCTCAAACATGTATCGGGTCTTACGGTCATTTTAAATCGCCCTGGAACCCCAATATTATGGGCAATAACTCATGAAAACGGCCATAAAACGCGAAAACGACGAGTTTTTGGTCATAACTCTCAAACATGTAAATCGCCTTGGAACCCCAAAACTATGGGCAATNGCTCATGAAAACGGCCATAAAACGCGAAAACGGNGAGTTTTTGGTCAT

*A. eriantha* centromeric satellite repeat (rnd-1_family-822#unknown)

TGATGCAGCCCAACACATGGNAATCACCATTGGTCCACATCATGCTACGCCAACCATNNCTACNAAGGTGAATTCCTTCCATCTCTTGCCTTCCTTTGGTCATCATGTGAATGGGATCCTACTTGAGACGAATNCCTATCGTATGACTAGGNCCATGAC
