# Supplementary material for: Genomic insights from the first chromosome-scale assemblies of oat (Avena spp.) diploid species
Source: BMC Biol. 2019 Nov 22;17:92. doi: 10.1186/s12915-019-0712-y (PMC6874827; doi:10.1186/s12915-019-0712-y)
Supplement: Supplementary file 3 — Additional file 3: Table S2. List of Avena accessions included in the resequencing panel. Cc 7277 (A. atlantica) and CN 19328 (A. eriantha) were the reference genomes. The species and genome formula of each accession is presented. The raw read files can be found in BioProject PRJNA556219. [file 12915_2019_712_MOESM3_ESM.docx]

**Additional file 3: Table S2.** List of Avena accessions included in the resequencing panel. Cc 7277 (*A. atlantica*) and CN 19328 (*A. eriantha*) were the reference genomes. The species and genome formula of each accession is presented. The raw read files can be found in BioProject PRJNA556219.

| **Accession^1^** | ***Avena* Taxon** | **BYU #** | **Genome Composition** | **Origin** | **Read Number** | **Total Gb** | **GC (%)^3^** | **SRR #^4^** |
| --- | --- | --- | --- | --- | --- | --- | --- | --- |
| CN 19328 | *eriantha* | 132 | C_p_C_p_ | Boghari, Algeria | 73084659 | 46.3 | 43.6 | SRR9933182 |
| CN 19256 | *eriantha* | 689 | C_p_C_p_ | Iran | 61475897 | 37.6 | 43.5 | SRR9933183 |
| CN 24022 | *eriantha* | 690 | C_p_C_p_ | Saida, Algeria | 75548151 | 47.1 | 43.3 | SRR9933180 |
| PI 657575 | *eriantha* | 765 | C_p_C_p_ | Ifrane, Morocco | 69247150 | 41.1 | 43.2 | SRR9933181 |
| PI 657576 | *eriantha* | 766 | C_p_C_p_ | Ifrane, Morocco | 70682569 | 42.4 | 43.3 | SRR9933178 |
| PI 657577 | *eriantha* | 767 | C_p_C_p_ | Ifrane, Morocco | 70519076 | 41.6 | 43.5 | SRR9933179 |
| PI 657578 | *eriantha* | 768 | C_p_C_p_ | Ifrane, Morocco | 72897296 | 43.0 | 43.4 | SRR9933138 |
| CN 19238 | *clauda* | 685 | C_p_C_p_ | Cardak, Turkey | 151146735 | 45.3 | 43.1 | SRR9933177 |
| CN 24040 | *clauda* | 686 | C_p_C_p_ | Batna, Algeria | 148442891 | 44.5 | 43.2 | SRR9933174 |
| unknown | *ventricosa* | 143 | C_v_C_v_ | unknown | 79861493 | 42.1 | 43.4 | SRR9933175 |
| CN 25888 | *atlantica* | 116 | A_s_A_s_ | Tiznit, Morocco | 154566346 | 46.4 | 44.1 | SRR9933163 |
| CN 25864 | *atlantica* | 139 | A_s_A_s_ | Tiznit, Morocco | 74048466 | 44.6 | 43.6 | SRR9933164 |
| CN 25849 | *atlantica* | 678 | A_s_A_s_ | Smimou, Morocco | 142044777 | 42.6 | 43.4 | SRR9933161 |
| CAV 6794 | *atlantica* | 680 | A_s_A_s_ | Tiznit, Morocco | 163834778 | 49.2 | 43.5 | SRR9933162 |
| Cc 7277 | *atlantica* | 803 | A_s_A_s_ | Wales | 74835030 | 46.4 | 43.4 | SRR9933159 |
| Cc 2043 | *strigosa brevis* | 202 | A_s_A_s_ | Unknown | 146455409 | 43.9 | 43.7 | SRR9933160 |
| CN 88826 | *strigosa brevis* | 681 | A_s_A_s_ | Portugal | 147799377 | 44.3 | 43.9 | SRR9933157 |
| PI 258545 | *strigosa brevis* | 804 | A_s_A_s_ | Portugal | 87790486 | 53.8 | 44.0 | SRR9933158 |
| PI 258544 | *strigosa brevis* | 807 | A_s_A_s_ | Portugal | 79377316 | 46.7 | 44.1 | SRR9933171 |
| PI 119009 | *strigosa brevis* | 808 | A_s_A_s_ | Rio Grande do Sul, Brazil | 70462076 | 42.6 | 43.6 | SRR9933172  SRR9933137 |
| CIav 9036 | *strigosa brevis* | 815 | A_s_A_s_ | St. Petersburg, Russia | 141858516 | 42.6 | 43.8 | SRR9933136 |
| PI 573533 | *strigosa brevis* | 825 | A_s_A_s_ | Portugal | 49219394 | 42.2 | 43.9 | SRR9933135 |
| CN 25698 | *strigosa hispanica* | 117 | A_s_A_s_ | Santa Eulalia, Portugal | 64245767 | 40.7 | 43.8 | SRR9933134 |
| CN 25675 | *strigosa hispanica* | 126 | A_s_A_s_ | Elvora, Portugal | 69517122 | 42.0 | 43.9 | SRR9933133 |
| CN 25727 | *strigosa hispanica* | 127 | A_s_A_s_ | Ponte de Sor, Portugal | 107456362 | 42.4 | 43.9 | SRR9933132 |
| CN 25788 | *strigosa hispanica* | 699 | A_s_A_s_ | Fataca, Portugal | 106978814 | 50.4 | 43.8 | SRR9933131 |
| PGR 10012 | *strigosa nuda* | 164 | A_s_A_s_ | Germany | 73560958 | 45.0 | 44.0 | SRR9933130 |
| PI 401830 | *strigosa nuda* | 809 | A_s_A_s_ | Germany | 71601935 | 43.1 | 43.5 | SRR9933129 |
| CIav 9009 | *strigosa nuda* | 812 | A_s_A_s_ | Canada, Ontario | 33263205 | 40.7 | 43.7 | SRR9933128 |
| PI 287319 | *strigosa nuda* | 826 | A_s_A_s_ | Nordrhein-Westfalen, Germany | 174893817 | 52.5 | 43.9 | SRR9933165 |
| PI 292226 | *strigosa* | 666 | A_s_A_s_ | Tel Aviv, Israel | 151996514 | 45.6 | 44.1 | SRR9933166 |
| PI 291990 | *strigosa* | 667 | A_s_A_s_ | Israel | 160623066 | 48.2 | 43.9 | SRR9933167 |
| CIav 9011 | *strigosa* | 668 | A_s_A_s_ | Denmark | 79103429 | 48.5 | 43.5 | SRR9933168 |
| CIav 9012 | *strigosa* | 669 | A_s_A_s_ | Bulgaria | 158464688 | 47.5 | 44.1 | SRR9933169 |
| CIav 9019 | *strigosa* | 670 | A_s_A_s_ | Wales, United Kingdom | 69501262 | 43.8 | 44.0 | SRR9933170 |
| CIav 9020 | *strigosa* | 671 | A_s_A_s_ | Argentina | 72377547 | 45.9 | 43.8 | SRR9933176 |
| CIav 9022 | *strigosa* | 672 | A_s_A_s_ | The Netherlands | 70391414 | 43.0 | 43.5 | SRR9933122 |
| CIav 9038 | *strigosa* | 673 | A_s_A_s_ | N. Ireland, United Kingdom | 160456988 | 48.1 | 43.9 | SRR9933126 |
| PI 186606 | *strigosa* | 790 | A_s_A_s_ | Rio Grande do Sul, Brazil | 74376194 | 44.1 | 43.9 | SRR9933143 |
| CIav 7010 | *strigosa* | 810 | A_s_A_s_ | Rio Grande do Sul, Brazil | 71708590 | 42.6 | 43.7 | SRR9933192 |
| CIav 9035 | *strigosa* | 814 | A_s_A_s_ | St. Petersburg, Russia | 131364137 | 39.4 | 43.6 | SRR9933191 |
| PI 83719 | *strigosa* | 816 | A_s_A_s_ | New South Wales, Australia | 141958189 | 42.6 | 43.6 | SRR9933194 |
| PI 83721 | *strigosa* | 817 | A_s_A_s_ | New South Wales, Australia | 127569408 | 38.3 | 43.6 | SRR9933193 |
| PI 244471 | *strigosa* | 818 | A_s_A_s_ | Rio Grande do Sul, Brazil | 147241840 | 44.2 | 43.8 | SRR9933196 |
| PI 244472 | *strigosa* | 819 | A_s_A_s_ | Rio Grande do Sul, Brazil | 145173656 | 43.6 | 43.7 | SRR9933195 |
| PI 304557 | *strigosa* | 820 | A_s_A_s_ | Wales, United Kingdom | 137039483 | 41.1 | 43.7 | SRR9933198 |
| PI 401800 | *strigosa* | 821 | A_s_A_s_ | Germany | 144664549 | 43.4 | 43.7 | SRR9933197 |
| PI 436031 | *strigosa* | 823 | A_s_A_s_ | La Araucania, Chile | 159527482 | 47.9 | 43.9 | SRR9933188 |
| PI 436102 | *strigosa* | 824 | A_s_A_s_ | La Araucania, Chile | 130424160 | 39.1 | 44.3 | SRR9933187 |
| CN 19731 | *hirtula* | 136 | A_s_A_s_ | El Asnam, Algeria | 84147361 | 47.6 | 44.0 | SRR9933151 |
| CN 19530 | *hirtula* | 179 | A_s_A_s_ | Antalya, Turkey | 157246047 | 47.2 | 43.6 | SRR9933152 |
| CN 21478 | *hirtula* | 691 | A_s_A_s_ | Aghia Varvara, Crete, Greece | 74717316 | 44.4 | 43.7 | SRR9933149 |
| CN 21653 | *hirtula* | 692 | A_s_A_s_ | Uras, Sardinia, Italy | 83735703 | 48.4 | 44.3 | SRR9933150 |
| CN 22965 | *hirtula* | 693 | A_s_A_s_ | Thibo, Tunisia | 83524397 | 45.9 | 43.6 | SRR9933155 |
| CN 25414 | *lusitanica* | 137 | A_s_A_s_ | Cordoba, Spain | 77332399 | 43.8 | 43.6 | SRR9933156 |
| CN 25777 | *lusitanica* | 140 | A_s_A_s_ | Senera, Portugal | 80342873 | 48.9 | 43.9 | SRR9933153 |
| CN 25807 | *lusitanica* | 141 | A_s_A_s_ | Ben Slimane, Morocco | 78489053 | 45 | 44.0 | SRR9933154 |
| CN 25843 | *lusitanica* | 142 | A_s_A_s_ | Essaouira, Morocco | 59464386 | 40.1 | 43.9 | SRR9933146 |
| CN 19342 | *wiestii* | 138 | A_s_A_s_ | Chalus, Iran | 80013311 | 45.6 | 43.9 | SRR9933147 |
| CN 24352 | *wiestii* | 178 | A_s_A_s_ | Misaf Hanegev, Israel | 70432801 | 43.1 | 43.8 | SRR9933125 |
| CN 19348 | *wiestii* | 696 | A_s_A_s_ | East Azerbaijan, Iran | 71135821 | 40.5 | 43.5 | SRR9933124 |
| CIav 1994 | *wiestii* | 801 | A_s_A_s_ | Giza, Egypt | 86330563 | 50.1 | 43.8 | SRR9933123 |
| CN 58138 | *longiglumis* | 149 | A_l_A_l_ | Oran, Algeria | 79480015 | 48.6 | 43.8 | SRR9933190 |
| CN 23032 | *canariensis* | 118 | A_c_A_c_ | Fuerteventura, Canary Is | 73878285 | 44.4 | 43.1 | SRR9933144 |
| CN 23029 | *canariensis* | 151 | A_c_A_c_ | Fuerteventura, Canary Is | 71771197 | 44.2 | 43.3 | SRR9933142 |
| CN 25449 | *canariensis* | 684 | A_c_A_c_ | Fuerteventura, Canary Is | 166741202 | 50 | 43.1 | SRR9933127 |
| CN19457 | *damascena* | 687 | A_d_A_d_ | Syria | 156187058 | 46.9 | 43.0 | SRR9933139 |
| CN19459 | *damascena* | 688 | A_d_A_d_ | Syria | 154286692 | 46.3 | 43.2 | SRR9933189 |
| PI 657458 | *damascena*^2^ | 740 | A_d_A_d_ | Ait Kemara, Morocco | 58619915 | 36.1 | 43.8 | SRR9933140 |
| PI 657471 | *damascena*^2^ | 743 | A_d_A_d_ | Laassara, Morocco | 73465434 | 40.3 | 43.8 | SRR9933186 |
| PI 657472 | *damascena*^2^ | 744 | A_d_A_d_ | Nador, Morocco | 66269179 | 40.8 | 43.5 | SRR9933145 |
| PI 657587 | *agadiriana* | 772 | A_1_A_1_A_2_A_2_ | Tifnit, Morocco | 71794451 | 42 | 43.2 | SRR9933141 |
| PI 657588 | *agadiriana* | 773 | A_1_A_1_A_2_A_2_ | Tiznit, Morocco | 79896522 | 47.1 | 43.3 | SRR9933185 |
| PI 657589 | *agadiriana* | 774 | A_1_A_1_A_2_A_2_ | Tiznit, Morocco | 69920058 | 43.8 | 43.4 | SRR9933173 |
| CIav 9008 | *sativa nuda* | 811 | AACCDD | Czechia | 67197168 | 38.6 | 43.5 | SRR9933148 |
| PI 401812 | *sativa nuda* | 822 | AACCDD | Germany | 137686083 | 41.3 | 43.3 | SRR9933184 |

^1^Origin: CI, CIav, and PI numbers are from the USDA-ARS National Plant Germplasm System (NPGS); CN and CAV numbers are from Plant Gene Resources of Canada (PGRC); PGR numbers are from the IPK-Gatersleben Gene Bank, Germany; Cc numbers are from the collection at the Institute of Biological, Environmental & Rural Sciences (IBERS), Aberystwyth University, Wales

^2^These three *A.* *damascena* accessions are likely misclassified based on the resequencing data

^3^GC content: (G & C base count) / (Total base count)

^4^NCBI Sequence read archive accessions number
