# Supplementary material for: Genomic insights from the first chromosome-scale assemblies of oat (Avena spp.) diploid species
Source: BMC Biol. 2019 Nov 22;17:92. doi: 10.1186/s12915-019-0712-y (PMC6874827; doi:10.1186/s12915-019-0712-y)
Supplement: Supplementary file 4 — Additional file 4: Table S3. SNPs per chromosome use for maximum likelihood phylogeny produced using SNPhylo [73]. [file 12915_2019_712_MOESM4_ESM.docx]

**Additional file 4: Table S3**. SNPs per chromosome use for maximum likelihood phylogeny produced using SNPhylo [72].

| Chromosome | Species (Accession) | Total Starting SNPs identified | Number of SNPs after pruning^1^ | % of SNPs after pruning |
| --- | --- | --- | --- | --- |
| AA1 | *A. atlantica* (Cc 7277) | 1,036,245 | 1,073 | 0.10% |
| AA2 | *A. atlantica* (Cc 7277) | 1,018,956 | 1,117 | 0.11% |
| AA3 | *A. atlantica* (Cc 7277) | 1,034,920 | 1,089 | 0.11% |
| AA4 | *A. atlantica* (Cc 7277) | 1,082,592 | 1,046 | 0.10% |
| AA5 | *A. atlantica* (Cc 7277) | 1,018,296 | 972 | 0.10% |
| AA6 | *A. atlantica* (Cc 7277) | 952,804 | 938 | 0.10% |
| AA7 | *A. atlantica* (Cc 7277) | 1,013,017 | 986 | 0.10% |
|  | *Total:* | 7,156,830 | 7,221 |  |
| AE1 | *A. eriantha* (CN 19328) | 3,725,125 | 1,724 | 0.05% |
| AE2 | *A. eriantha* (CN 19328) | 4,392,742 | 1,756 | 0.04% |
| AE3 | *A. eriantha* (CN 19328) | 3,891,851 | 1,726 | 0.04% |
| AE4 | *A. eriantha* (CN 19328) | 3,526,546 | 1,690 | 0.05% |
| AE5 | *A. eriantha* (CN 19328) | 3,826,401 | 1,666 | 0.04% |
| AE6 | *A. eriantha* (CN 19328) | 3,493,915 | 1,479 | 0.04% |
| AE7 | *A. eriantha* (CN 19328) | 4,259,350 | 1,489 | 0.03% |
|  | *Total:* | 27,115,930 | 11,530 |  |

^1^SNPs were pruned using a linkage disequilbrium of 0.1, a missing rate > 0.1 using a 500,000 base pair sliding window
