# Supplementary figures and images for: Genomic insights from the first chromosome-scale assemblies of oat (Avena spp.) diploid species
Source: BMC Biol. 2019 Nov 22;17:92. doi: 10.1186/s12915-019-0712-y (PMC6874827; doi:10.1186/s12915-019-0712-y)

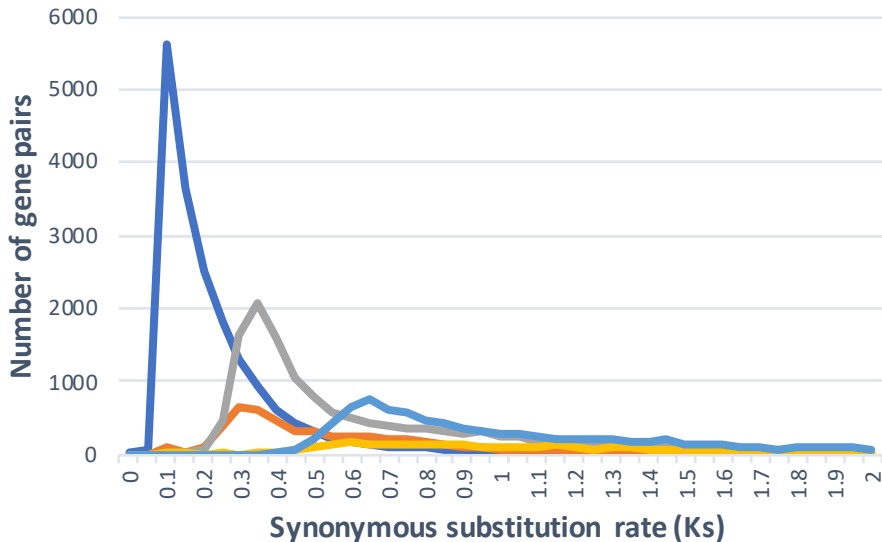

A. eriantha

H. vulgare

B. distachyon

O. sativa

Z. mays

Supplement: Supplementary file 5 — Additional file 5: Figure S1. Rate of synonymous substitutions per synonymous sites (Ks) within duplicated gene pairs from coding sequences predicted from A. atlantica comparisons with A. eriantha, H. vulgare, B. distachyon, O. sativa, and Z. mays. [file 12915_2019_712_MOESM5_ESM.pdf]

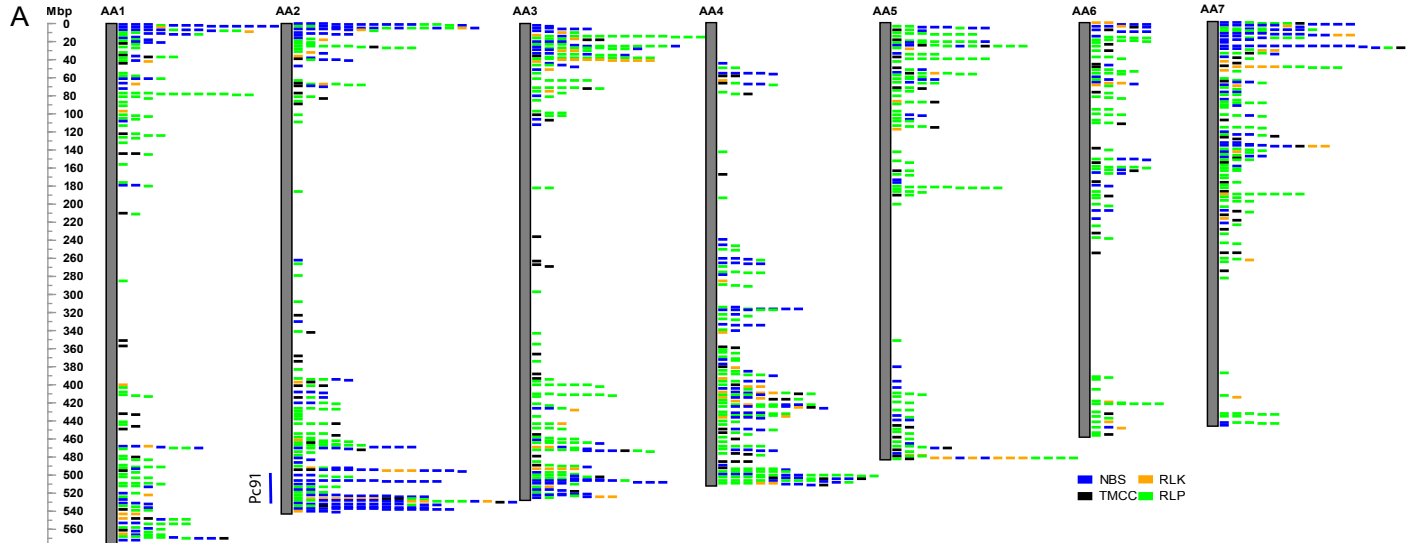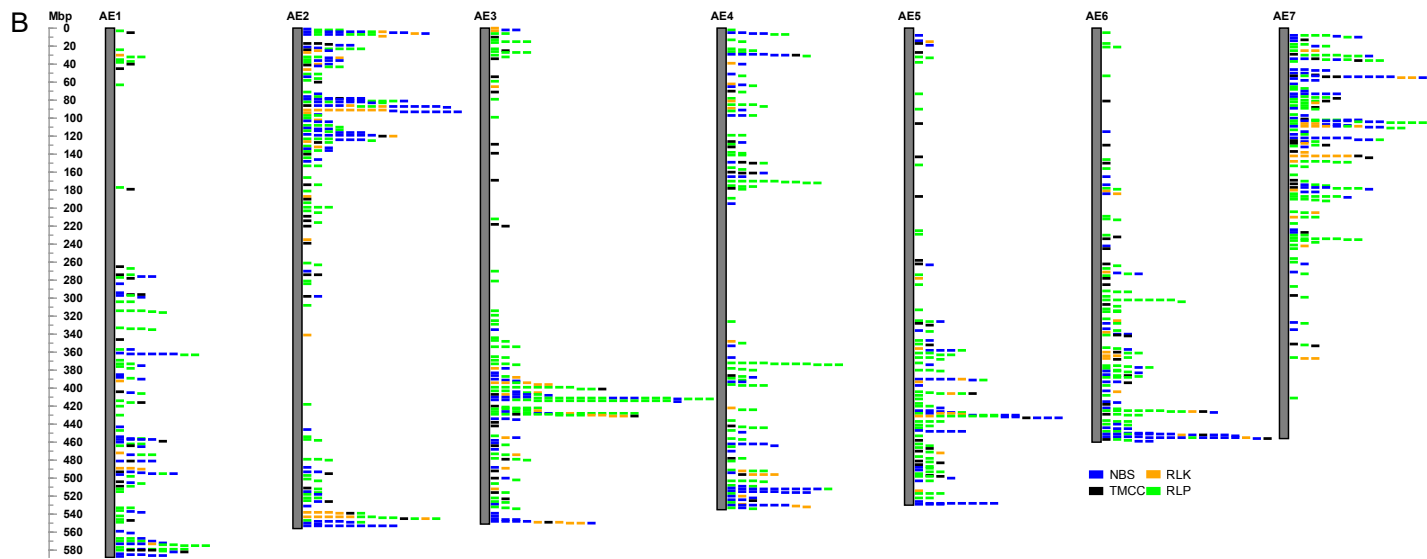

Supplement: Supplementary file 10 — Additional file 10: Figure S5. Distribution of the resistance gene analogs (RGAs) encoding genes on the (A) A. atlantica and (B) A. eriantha genome. The RGAugury pipeline classifies RGA candidates into four major families based on the presence of RGA domains and motifs, specifically, nucleotide binding sites (NBS, blue), transmembrane coiled-coil (TMCC, black), and membrane associated receptor-like proteins kinases (RLK, yellow) and receptor-like proteins (RLP, green). The predicted location of the Pc91 crown rust QTLs (Klos et al. [30]) in the A. atlantica genome is shown on chromosome AA2. [file 12915_2019_712_MOESM10_ESM.pdf]

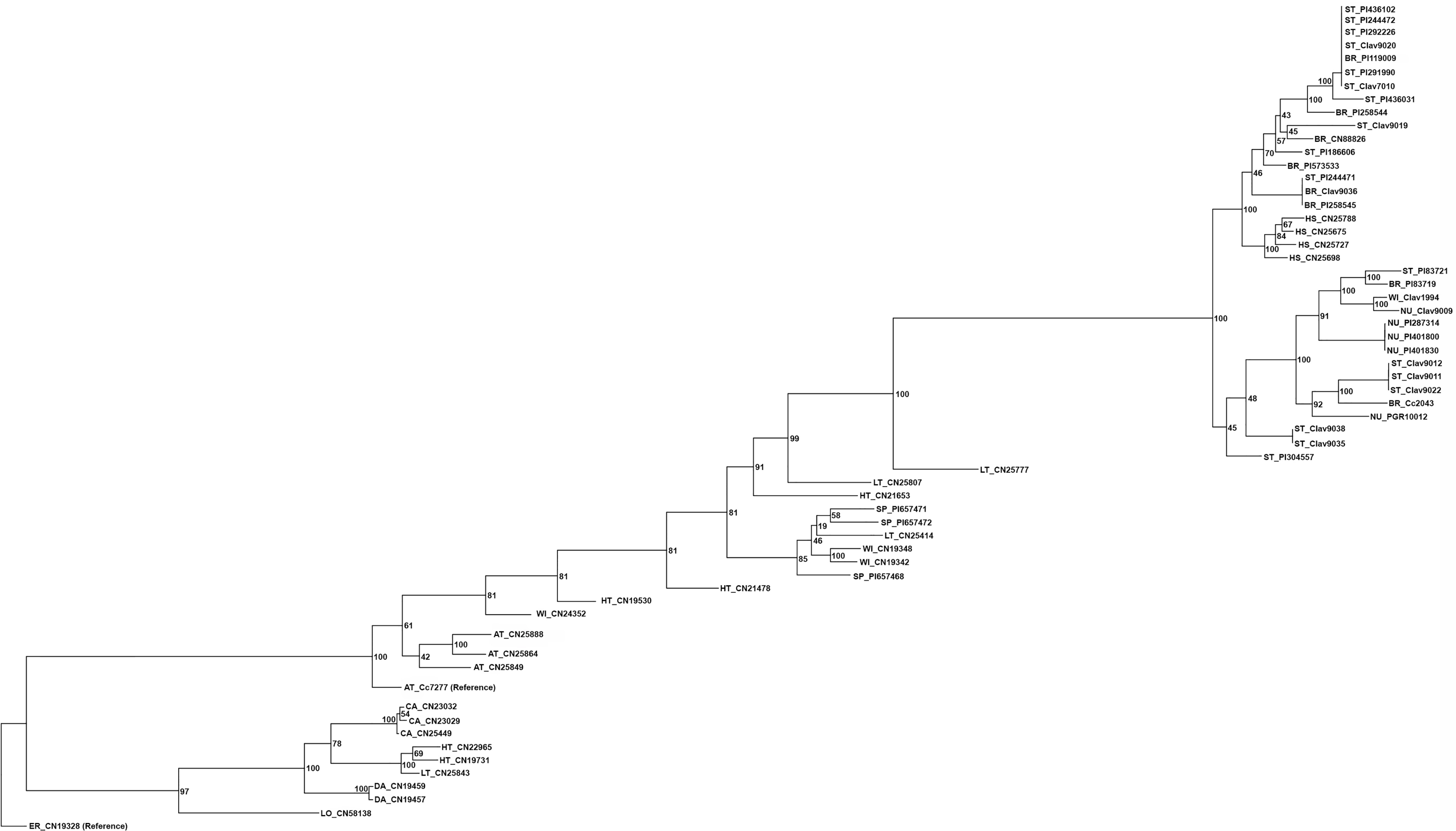

Supplement: Supplementary file 15 — Additional file 15: Figure S7. Unabbreviated A-genome diploids rooted to the A. eriantha reference (ER_CN 19238). Accession names are abbreviated as described in Additional file 3: Table S2. [file 12915_2019_712_MOESM15_ESM.pdf]

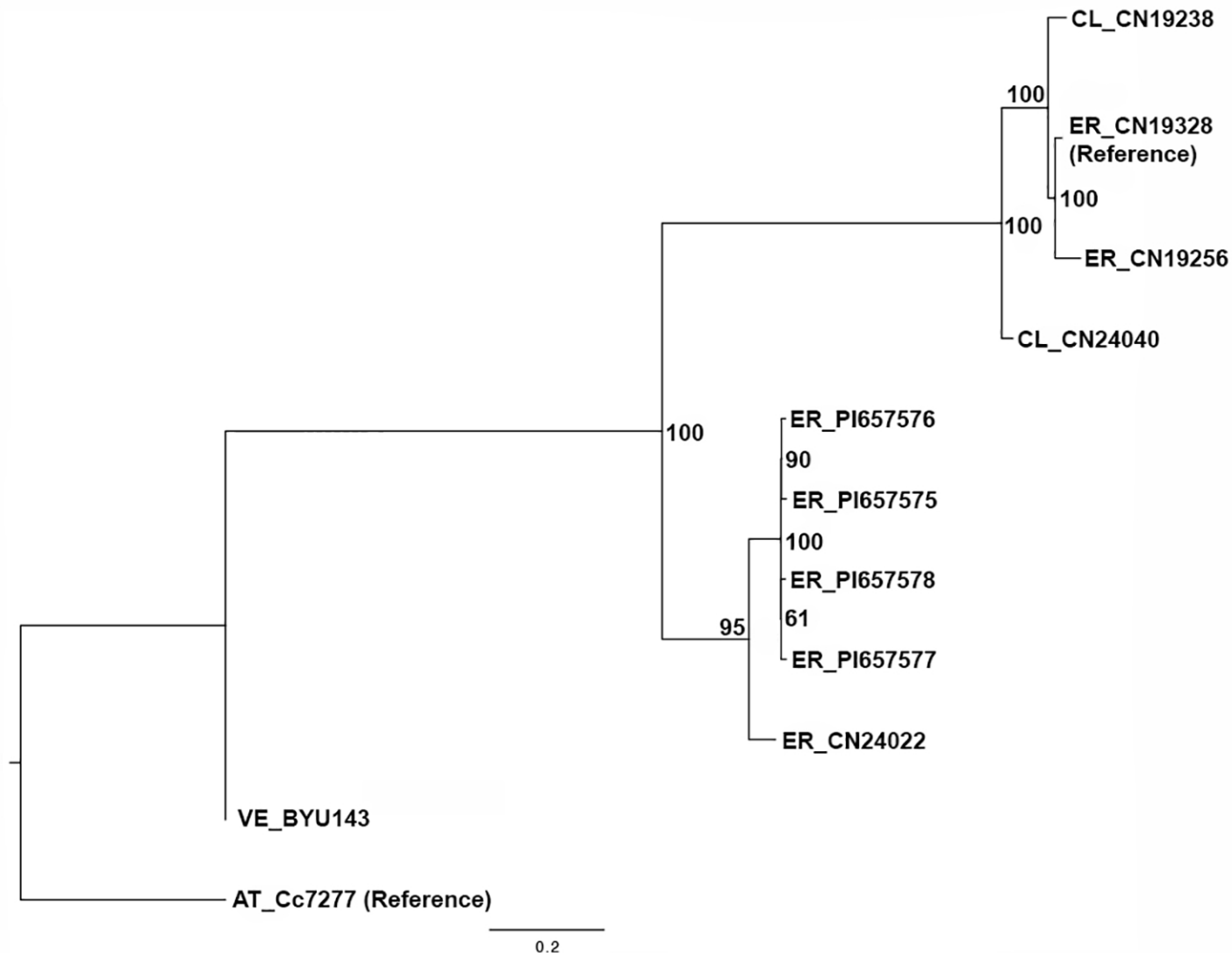

Supplement: Supplementary file 16 — Additional file 16: Figure S8. Unabbreviated C-genome diploids rooted to the A. atlantica (AT_Cc 7277) reference. Accession names are abbreviated as described in Additional file 3: Table S2. [file 12915_2019_712_MOESM16_ESM.pdf]
