# Supplementary material for: Genomic insights from the first chromosome-scale assemblies of oat (Avena spp.) diploid species
Source: BMC Biol. 2019 Nov 22;17:92. doi: 10.1186/s12915-019-0712-y (PMC6874827; doi:10.1186/s12915-019-0712-y)
Supplement: Supplementary file 6 — Additional file 6: Figure S2. Orthologous genes were identified between A. atlantica and A. eriantha genomes to detect orthologous chromosome relationships. Genome synteny was (A) visualized by dotplot analysis, with boxes drawn around syntenic regions, (B) quantified, where the chromosome pairs with the highest amount of syntenic block connections, expressed as a percentage of the total syntenic bases, are colored red and transition to white as the number of connections decreases and (C) correlation between syntenic block sizes between A. atlantica and A. eriantha. [file 12915_2019_712_MOESM6_ESM.pdf]

A

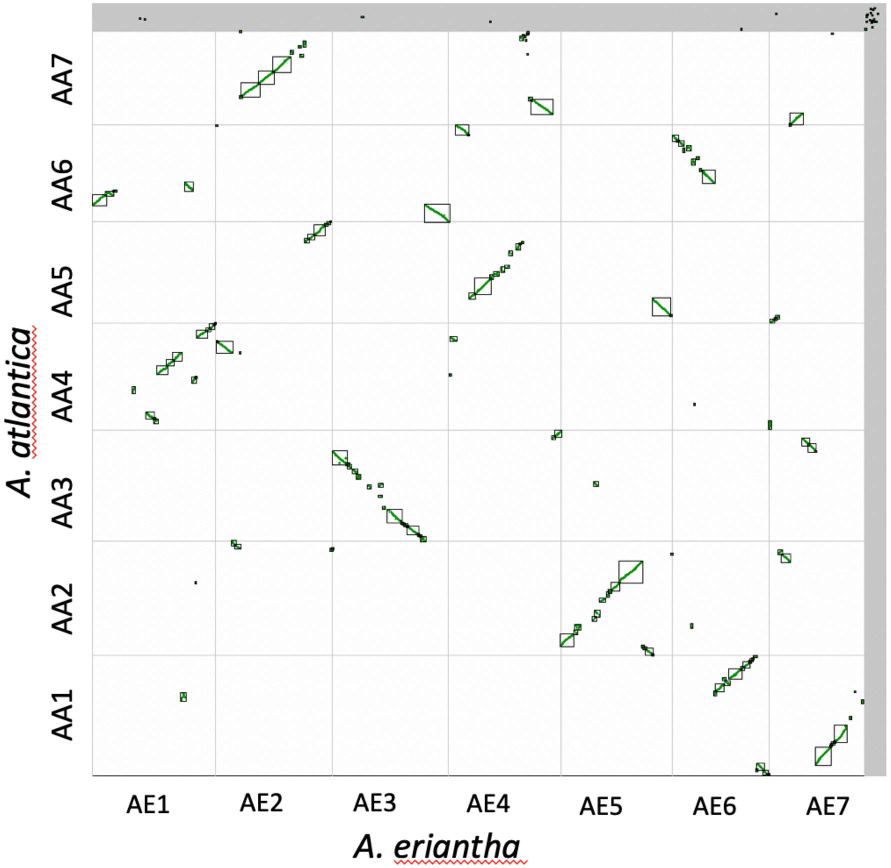

C

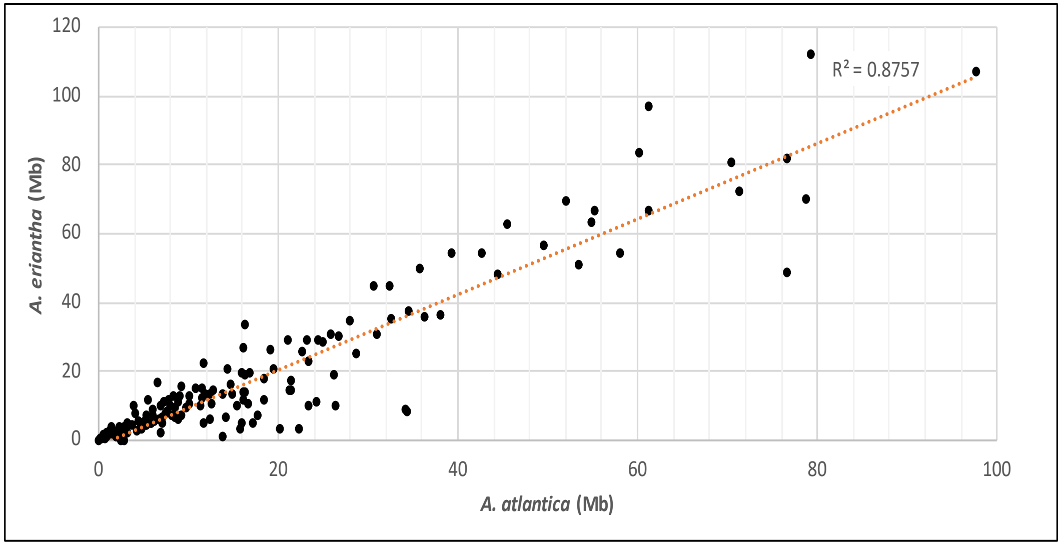

B

A. atlantica Hi-C scaffold/Chromosome name		*A. eriantha* Hi-C scaffold/Chromosome Name														Total syntenic sequence (bp)	Orthologs†
		1585_3068		4_4		2055_3936		430_889		1285_2510		670_1352		2331_4482			
		**AE6**		**AE5**		**AE3**		**AE1**		**AE4**		**AE7**		**AE2**			
324_449	**AA1**	61%	248,113,104	0%	-	0%	-	2%	8,964,879	0%	-	37%	150,069,527	0%	-	407,147,510	AE6 (60%); AE7 (36%)
1702_2338	**AA2**	1%	6,268,489	77%	349,869,485	0%	-	0%	-	0%	-	11%	50,572,926	10%	46,620,358	453,331,258	AE5 (77%)
2069_2732	**AA3**	0%	-	3%	14,026,198	74%	317,581,481	0%	-	10%	43,128,960	13%	55,964,464	0%	-	430,701,103	AE3 (74%)
350_483	**AA4**	1%	2,448,066	0%	-	0%	-	71%	271,866,573	8%	28,837,464	2%	7,950,910	19%	72,738,363	383,841,376	AE1 (71%)
1310_1788	**AA5**	0%	-	22%	85,602,020	0%	-	0%	-	41%	158,055,695	9%	34,177,532	28%	109,867,904	387,703,151	AE4 (41%); AE2 (28%)
2050_2712	**AA6**	29%	128,665,157	0%	-	25%	111,842,443	33%	146,006,081	13%	56,972,331	0%	-	0%	1,603,520	445,089,532	AE1 (32%); AE6 (29%); AE3 (25%)
1760_2399	**AA7**	0%	-	0%	-	0%	-	0%	-	29%	139,831,412	13%	64,550,926	57%	274,094,733	478,477,071	AE2 (57%); AE4 (29%)

[1]Subgenome orthologous chromosomes representing > 50% syntenic blocks.
