# Supplementary material for: Genomic insights from the first chromosome-scale assemblies of oat (Avena spp.) diploid species
Source: BMC Biol. 2019 Nov 22;17:92. doi: 10.1186/s12915-019-0712-y (PMC6874827; doi:10.1186/s12915-019-0712-y)
Supplement: Supplementary file 7 — Additional file 7: Figure S3. Orthologous genes were identified between A. atlantica and H. vulgare genomes to detect orthologous chromosome relationships. Genome synteny was (A) visualized by dotplot analysis, with boxes drawn around syntenic regions, (B) quantified, where the chromosome pairs with the highest amount of syntenic block connections, expressed as a percentage of the total syntenic bases, are colored red and transition to white as the number of connections decreases and (C) correlation between syntenic block sizes between A. atlantica and H. vulgare (Hv_IBSC_PGSB_v2; Ensembl Release 36). [file 12915_2019_712_MOESM7_ESM.pdf]

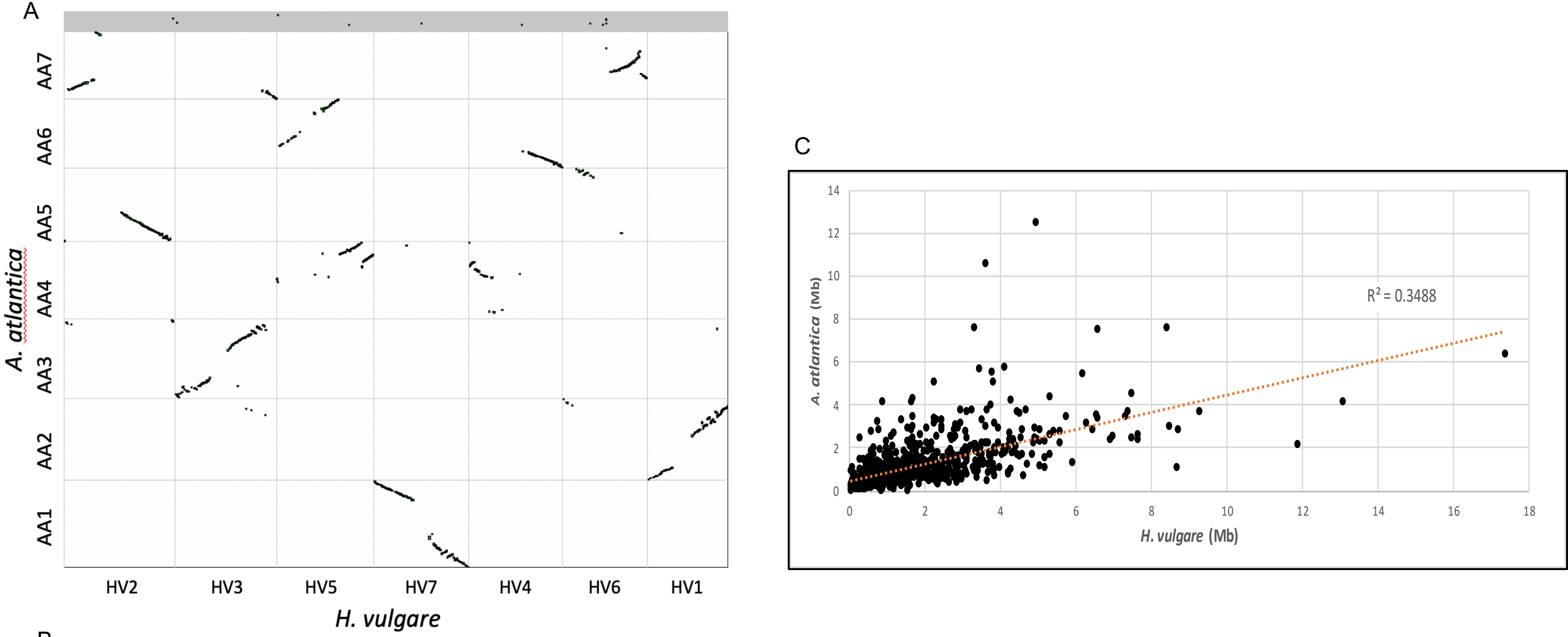


B

| *H. vulgare* chromosomes | *A. atlantica* chromosome | | | | | | | | | | | | | | Total syntenic sequence (bp) |
|---|---|---|---|---|---|---|---|---|---|---|---|---|---|---|---|
| | AA1 | | AA2 | | AA3 | | AA4 | | AA5 | | AA6 | | AA7 | | |
| **HV1** | 0% | - | 99% | 116,552,573 | 1% | 827,377 | 0% | - | 0% | - | 0% | - | 0% | - | 117,379,950 |
| **HV2** | 0% | - | 0% | - | 3% | 3,970,402 | 0% | - | 69% | 93,678,206 | 0% | - | 29% | 38,970,738 | 136,619,346 |
| **HV3** | 0% | - | 2% | 2,583,749 | 88% | 131,236,323 | 0% | - | 0% | - | 0% | - | 10% | 15,378,574 | 149,198,646 |
| **HV4** | 0% | - | 0% | - | 0% | - | 41% | 42,236,801 | 0% | - | 59% | 61,862,029 | 0% | - | 104,098,830 |
| **HV5** | 0% | - | 0% | - | 0% | - | 54% | 72,599,961 | 0% | - | 46% | 62,333,988 | 0% | - | 134,933,949 |
| **HV6** | 0% | - | 5% | 5,476,359 | 0% | - | 0% | - | 22% | 25,316,901 | 0% | - | 73% | 82,857,940 | 113,651,200 |
| **HV7** | 99.7% | 125,071,153 | 0% | - | 0% | - | 0% | 409,703 | 0% | - | 0% | - | 0% | - | 125,480,856 |
| **Orthologs[1]** | HV7 (99.7%) | | HV1 (99%) | | HV3 (88%) | | HV5 (54%); HV4 (41%) | | HV2 (69%) | | HV4 (59%); HV5 (46%) | | HV6 (73%) | | |

[1]Subgenome orthologous chromosomes representing > 50% syntenic blocks.
