# Supplementary material for: Genomic insights from the first chromosome-scale assemblies of oat (Avena spp.) diploid species
Source: BMC Biol. 2019 Nov 22;17:92. doi: 10.1186/s12915-019-0712-y (PMC6874827; doi:10.1186/s12915-019-0712-y)
Supplement: Supplementary file 8 — Additional file 8: Figure S4. Orthologous genes were identified between A. eriantha and H. vulgare genomes to detect orthologous chromosome relationships. Genome synteny was (A) visualized by dotplot analysis, with boxes drawn around syntenic regions, (B) quantified, where the chromosome pairs with the highest amount of syntenic block connections, expressed as a percentage of the total syntenic bases, are colored red and transition to white as the number of connections decreases and (C) correlation between syntenic block sizes between A. eriantha and H. vulgare (Hv_IBSC_PGSB_v2; Ensembl Release 36). [file 12915_2019_712_MOESM8_ESM.pdf]

A

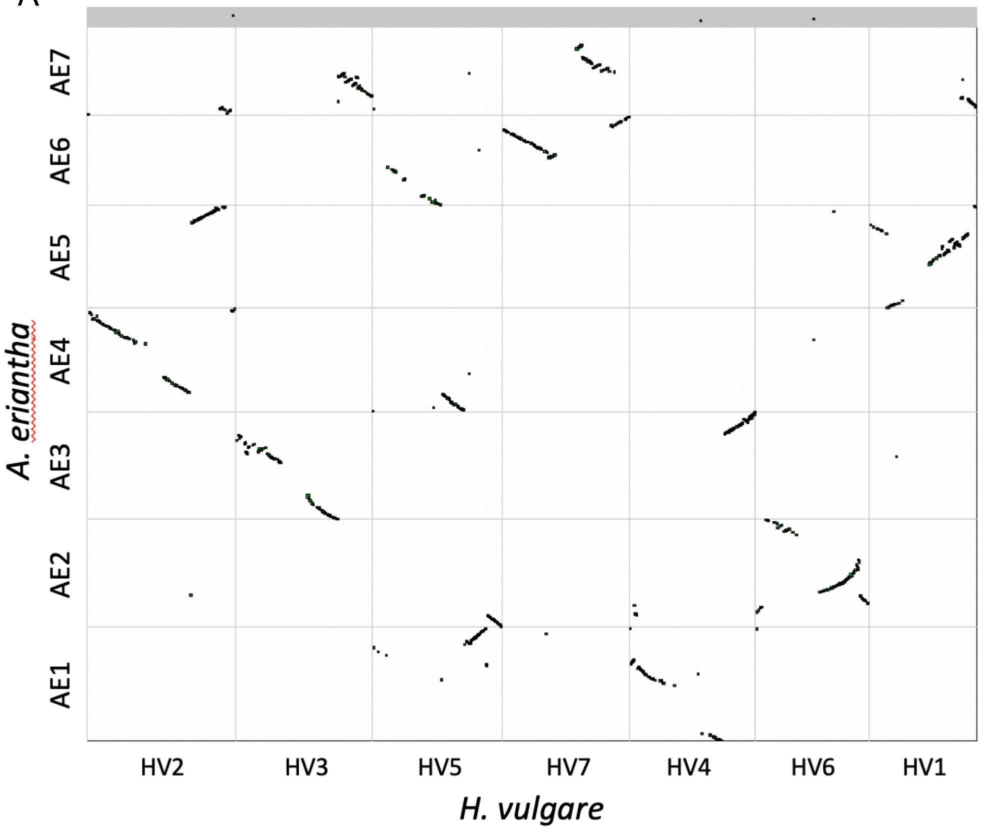

C

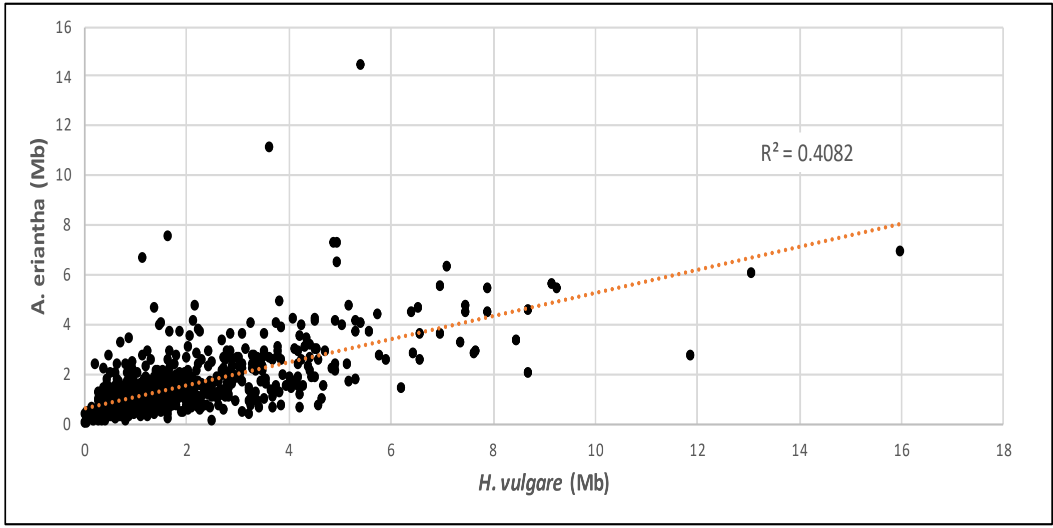

B

H. vulgare chromosomes	*A. eriantha* chromosome														Total syntenic sequence (bp)
	AE1		AE2		AE3		AE4		AE5		AE6		AE7		
**HV1**	0%	-	0%	-	0%	155,397	0%	-	85%	105,383,642	0%	-	15%	18,862,612	124,401,651
**HV2**	0%	-	0%	701,399	0%	-	65%	113,211,874	28%	49,394,789	0%	-	6%	10,493,315	173,801,377
**HV3**	0%	-	0%	-	64%	106,439,135	0%	-	0%	-	0%	-	36%	58,708,358	165,147,493
**HV4**	52%	78,704,051	4%	6,618,174	44%	66,297,455	0%	-	0%	-	0%	-	0%	-	151,619,680
**HV5**	30%	44,906,428	15%	22,615,266	0%	-	28%	42,982,878	0%	-	27%	41,512,321	0%	36,184	152,053,077
**HV6**	1%	1,002,263	98%	139,586,141	0%	-	0%	44,332	1%	1,150,999	0%	-	0%	-	141,783,735
**HV7**	0%	603,748	0%	-	0%	-	0%	-	0%	-	53%	71,356,066	47%	63,613,086	135,572,900
**Orthologs[1]**	HV4 (52%)		HV6 (98%)		HV3 (64%)		HV2 (65%)		HV1 (85%)		HV7 (53%)		-		

[1]Subgenome orthologous chromosomes representing > 50% syntenic blocks.
