# Supplementary material for: Genomic insights from the first chromosome-scale assemblies of oat (Avena spp.) diploid species
Source: BMC Biol. 2019 Nov 22;17:92. doi: 10.1186/s12915-019-0712-y (PMC6874827; doi:10.1186/s12915-019-0712-y)
Supplement: Supplementary file 9 — Additional file 9: Table S4. Summary of resistance gene analog identification results using the RGAugury pipeline [92] for A. atlantica and A. eriantha. [file 12915_2019_712_MOESM9_ESM.docx]

**Additional file 9: Table S4**. Summary of resistance gene analog identification results using the RGAugury pipeline [91] for *A. atlantica* and *A. eriantha*.

| Species | Protein sequences | Mean Protein length (aa) | NBS encoding^1^ | | | | | | | | RLP | RLK | TM-CC | Total |
| --- | --- | --- | --- | --- | --- | --- | --- | --- | --- | --- | --- | --- | --- | --- |
|  |  |  | NBS | CC-NBS-LRR | TIR-NBS-LRR | CC-NBS | TIR-NBS | NBS-LRR | TIR-unknown | Others |  |  |  |  |
| *A. atlantica* | 49,542 | 369 | 45 | 226 | 0 | 40 | 2 | 195 | 3 | 0 | 120 | 772 | 160 | 1563 |
| *A. eriantha* | 47,361 | 346 | 50 | 190 | 0 | 42 | 1 | 174 | 2 | 0 | 135 | 654 | 154 | 1402 |
| ^1^CC: Coiled-coil; LRR: Leucine rich repeat; NBS: Nucleotide-binding site; RLP: Receptor like protein; RLK: Receptor like kinase; TIR: Toll/Interleukin-1 receptor; TM: Transmembrane | | | | | | | | | | | | | | |
