# Supplementary material for: Genomic insights from the first chromosome-scale assemblies of oat (Avena spp.) diploid species
Source: BMC Biol. 2019 Nov 22;17:92. doi: 10.1186/s12915-019-0712-y (PMC6874827; doi:10.1186/s12915-019-0712-y)
Supplement: Supplementary file 11 — Additional file 11: Table S5. Candidate resistance gene analogs associated with crown rust resistance on Mrg18 linkage group [28]. Mrg18 was previously shown to be involved in an intergenomic translocation involving 7C and 17A, corresponding to A. eriantha chromosome AE7 and A. atlantica chromosome AA2. Klos et al. [30] identified two QTLs associated P. coronata (crown rust) resistance on Mrg18, one of which determined to be Pc91. Candidate resistance gene analogs were identified using BLAST searches against the A. atlantica and A. eriantha genome assembly using makers sequences associated with the QTLs. [file 12915_2019_712_MOESM11_ESM.docx]

**Additional file 11: Table S5**. Candidate resistance gene analogs associated with crown rust resistance on Mrg18 linkage group [28]. Mrg18 was previously shown to be involved in an intergenomic translocation involving 7C and 17A, corresponding to *A. eriantha* chromosome AE7 and *A. atlantica* chromosome AA2. Klos et al. [30] identified two QTLs associated *P. coronata* (crown rust) resistance on Mrg18, one of which determined to be Pc91. Candidate resistance gene analogs were identified using BLAST searches against the *A. atlantica* and *A. eriantha* genome assembly using makers sequences associated with the QTLs.

| QTL | Resistance gene | SNP name | Linkage group | cM position | Chr Origin | Chr | Marker position | E-value | Mapping species^1^ | Closest RGA | RGA Type | Annotation ID^2^ |
| --- | --- | --- | --- | --- | --- | --- | --- | --- | --- | --- | --- | --- |
| QPc.CORE.18.1 | NA | GMI_DS_  LB_2908 | Mrg18 | 21.1 | C/A | AE7 | 70,260,284 | 1E-103 | AE | 70,245,021 | NBS-NL | AE028733 |
| QPc.CORE.18.2 | *Pc91* | GMI_ES03_  c2277_336 | Mrg18 | 67.7 | C/A | AA2 | 496,455,574 | 1E-57 | AA | 496,480,229 | NBS-NL | AA013068 |
| QPc.CORE.18.3 | *Pc91* | GMI_ES05_  c11155_383 | Mrg18 | 67.7 | C/A | AA2 | 533,475,317 | 5E-53 | AA | 533,698,614 | NBS-CNL | AA014151 |
| QPc.CORE.18.3 | *Pc91* | GMI_GBS_  24408 | Mrg18 | 67.7 | C/A | AA2 | 510,519,361 | 5E-25 | AA | 510,828,316 | NBS-CNL | AA013376 |

^1^AE = *A. eriantha*; AA = *A. atlantica*

^2^Annotation description: AE028733 = Similar to RPM1: Disease resistance protein RPM1 (*A. thaliana*); AA013068 = Similar to RGA3: Putative disease resistance protein RGA3 (*Solanum bulbocastanum*); AA014151 = Similar to RPM1: Disease resistance protein RPM1 (*A. thaliana*); AA013376 =Similar to RPH8A: Disease resistance protein RPH8A (*A. thaliana*)
