# Supplementary material for: Genomic insights from the first chromosome-scale assemblies of oat (Avena spp.) diploid species
Source: BMC Biol. 2019 Nov 22;17:92. doi: 10.1186/s12915-019-0712-y (PMC6874827; doi:10.1186/s12915-019-0712-y)
Supplement: Supplementary file 12 — Additional file 12: Figure S6. Corresponding location of restriction fragment length polymorphism markers mapped on a segregating A. strigosa X A. wiestii population developed by Kremer et al. [102] on the A. atlantica chromosomes (shown on a gene density plot). Markers from each of their linkage groups (Asw A-I) are color-coded with approximate positions on A. atlantica scaffolds indicated with arrows. [file 12915_2019_712_MOESM12_ESM.pdf]

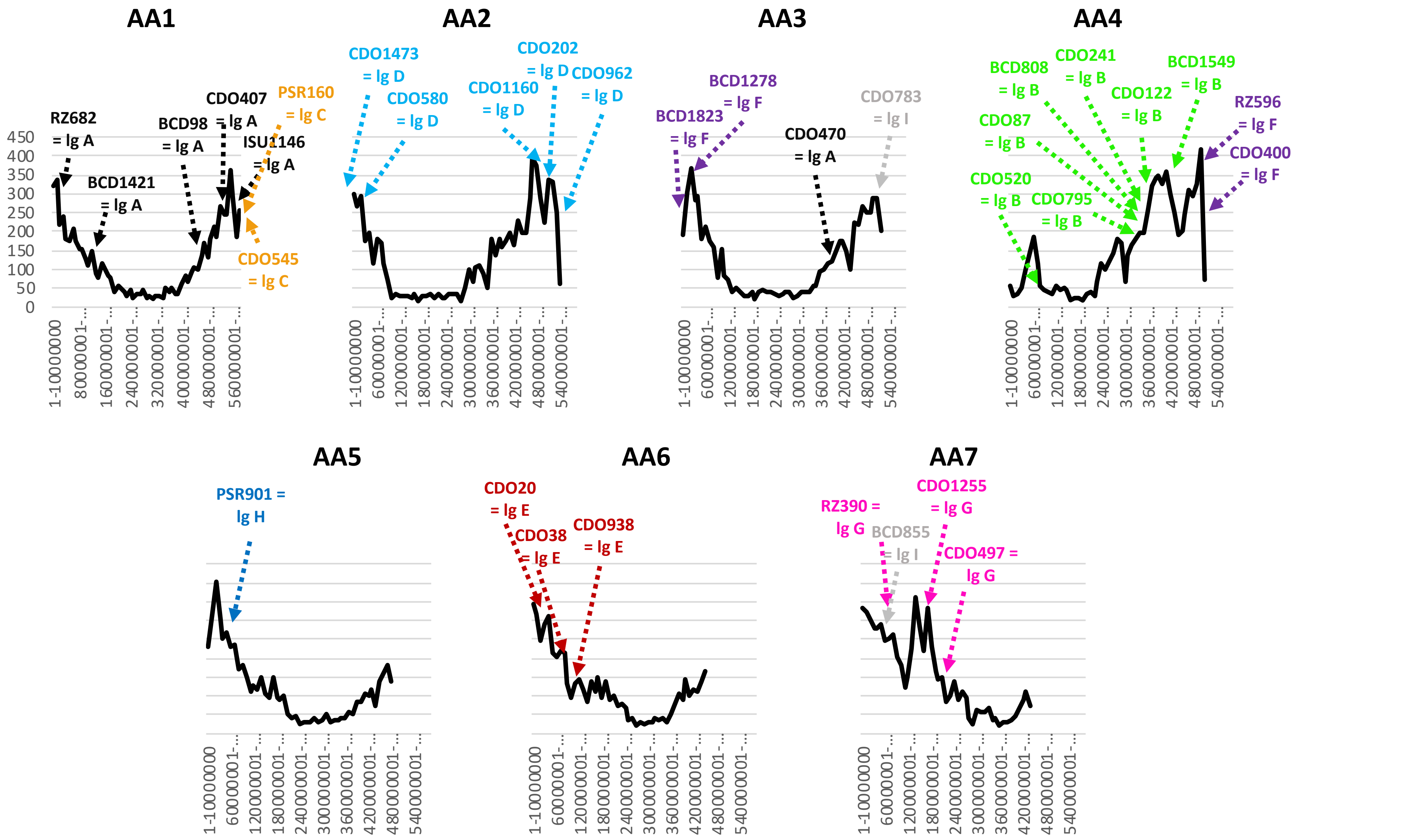

Linkage group + *A. atlantica* chromosome assignments

AswA = AA1 + AA3 | *AswB* = AA4 | *AswC* = AA1 | *AswD* = AA2

AswE = AA6 | *AswF* = AA3+ AA4 | *AswG* = AA7 | *AswH* = AA5

AswI = undefined
