# Supplementary material for: Genomic insights from the first chromosome-scale assemblies of oat (Avena spp.) diploid species
Source: BMC Biol. 2019 Nov 22;17:92. doi: 10.1186/s12915-019-0712-y (PMC6874827; doi:10.1186/s12915-019-0712-y)
Supplement: Supplementary file 13 — Additional file 13: Table S6. PacBio and Illumina sequencing read statistics. The raw read files can be found in BioProjects PRJNA546592 and PRJNA546595. [file 12915_2019_712_MOESM13_ESM.docx]

**Additional file 13: Table S6.** PacBio and Illumina sequencing read statistics. The raw read files can be found in BioProjects PRJNA546592 and PRJNA546595.

| **Species** | **Accession** | **Technology** | **Number of Reads** | **Total Gb** | **Longest Read (bp)** | **Mean Read (bp)** | **Read N50 (bp)** | **Genome Coverage** | |
| --- | --- | --- | --- | --- | --- | --- | --- | --- | --- |
| *A. atlantica* | Cc 7277 | Sequel | 23,475,393 | 246.6 | 194,884 | 10,706 | 18,242 | | 66.6X |
| *A. atlantica* | Cc 7277 | RS II^1^ | 7,737,947 | 75.3 | 76,481 | 9,432 | 17,317 | | 20.4X |
| *A. atlantica* | Cc 7277 | RS II^2^ | 331,056 | 4.0 | 74,575 | 12,373 | 20,414 | | 1.1X |
| *A. eriantha* | CN 19328 | Sequel | 28,257,346 | 276.6 | 151,576 | 8,699 | 15,102 | | 65.9X |

^1^Sequencing performed by the Arizona Genomics Institute, University of Arizona, Tucson, AZ

^2^Sequencing performed by the RTL Genomics, Lubbock, TX
