# Supplementary material for: Genomic insights from the first chromosome-scale assemblies of oat (Avena spp.) diploid species
Source: BMC Biol. 2019 Nov 22;17:92. doi: 10.1186/s12915-019-0712-y (PMC6874827; doi:10.1186/s12915-019-0712-y)
Supplement: Supplementary file 14 — Additional file 14: Table S7. Raw read statistics for RNASeq data for A. atlantica and A. eriantha. All reads were illumina pair-end reads from standard 500 bp insert libraries. The raw read files can be found in BioProjects PRJNA556794 and PRJNA546595. [file 12915_2019_712_MOESM14_ESM.docx]

**Additional file 14: Table S7.** Raw read statistics for RNASeq data for *A. atlantica* and *A. eriantha*. All reads were Illumina pair-end reads from standard 500 bp insert libraries. The raw read files can be found in BioProjects PRJNA556794 and PRJNA546595.

| **Source** | **Species (accession)** | **Read Length (bp)** | **No. of Reads** | **Total bp** |
| --- | --- | --- | --- | --- |
| Stem | *A. atlantica* (Cc 7277) | 101 | 16,597,324 | 1,676,329,724 |
| Mature leaf | *A. atlantica* (Cc 7277) | 101 | 23,773,832 | 2,401,157,032 |
| Stressed Mature leaf | *A. atlantica* (Cc 7277) | 101 | 23,937,698 | 2,417,707,498 |
| Seed (2 days old) | *A. atlantica* (Cc 7277) | 101 | 23,451,368 | 2,368,588,168 |
| Hypocotyl (4/5 day old) | *A. atlantica* (Cc 7277) | 101 | 30,204,274 | 3,050,631,674 |
| Root (4/5 days old) | *A. atlantica* (Cc 7277) | 101 | 24,402,346 | 2,464,636,946 |
| Vegetative meristem | *A. atlantica* (Cc 7277) | 101 | 23,448,778 | 2,368,326,578 |
| Green grain | *A. atlantica* (Cc 7277) | 101 | 28,358,800 | 2,864,238,800 |
| Yellow grain | *A. atlantica* (Cc 7277) | 101 | 22,643,900 | 2,287,033,900 |
| Young flower (meiotic) | *A. atlantica* (Cc 7277) | 101 | 22,817,776 | 2,304,595,376 |
| Green anthers | *A. atlantica* (Cc 7277) | 101 | 25,725,050 | 2,598,230,050 |
| Young leaf | *A. eriantha* (CN 19328) | 150 | 67,538,458 | 10,130,768,700 |
| Mature leaf | *A. eriantha* (CN 19328) | 150 | 52,218,112 | 7,832,716,800 |
| Crown tissue | *A. eriantha* (CN 19328) | 150 | 55,027,004 | 8,254,050,600 |
| Roots | *A. eriantha* (CN 19328) | 150 | 60,794,380 | 9,119,157,000 |
| Whole seedling | *A. eriantha* (CN 19328) | 150 | 64,350,142 | 9,652,521,300 |
| Immature panicle | *A. eriantha* (CN 19328) | 150 | 68,747,494 | 10,312,124,100 |
